# Evaluating Force Matching as a Parametrization Strategy for the CHARMM36m Force Field Using Phosphorylation

**DOI:** 10.1021/acs.jpcb.6c00779

**Published:** 2026-05-22

**Authors:** Viktoria Korn, Tobias Rindfleisch, Sandra Posch, Jarl Underhaug, Andreas Horner, Markus Miettinen, Kristyna Pluhackova

**Affiliations:** † Stuttgart Center for Simulation Science, Cluster of Excellence EXC 2075, 9149 University of Stuttgart, Stuttgart 70569, Germany; ‡ Computational Biology Unit, Department of Informatics, 1658 University of Bergen, Bergen 5008, Norway; ¶ Department of Chemistry, 1658 University of Bergen, Bergen 5007, Norway; § Max Planck Institute of Molecular Plant Physiology, Potsdam 14476, Germany; || Institute of Biophysics, 27266 Johannes Kepler University Linz, Linz 4040, Austria

## Abstract

Phosphorylation is a central regulatory post-translational modification whose accurate representation is essential for molecular simulations of biomolecular systems. Although CHARMM includes parameters for phosphorylated residues, their nonbonded interactions were largely estimated decades ago and have remained insufficiently validated due to the scarcity of experimental data for highly charged phosphate groups and the inherent chemical instability of the phosphoester linkage in model compounds. Here, we reparametrize the amino acid side-chain analog methylphosphate, a model for phosphorylated serine, in multiple charge states using force matching to density functional theory reference data obtained from our own quantum chemical calculations in an aqueous environment. The resulting parameters are validated against new experimental measurements, including osmotic pressure measured as osmotic concentration and nuclear magnetic resonance (NMR) relaxation data for phosphorylated dipeptides. This combined computational–experimental approach enables a systematic refinement of the nonbonded parameters for all relevant phosphate charge states, yielding physically accurate hydration and ion-interaction behavior while maintaining compatibility with the CHARMM36m force field.

## Graphical abstract

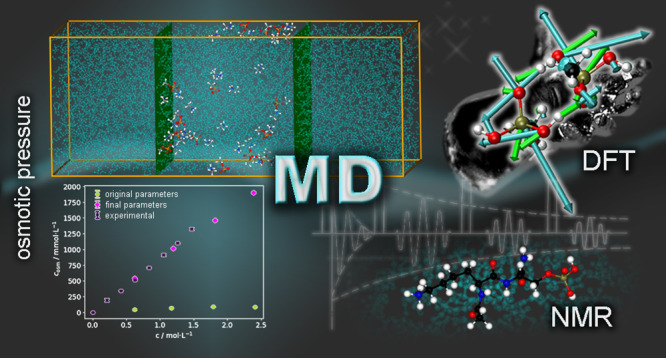

## 1 Introduction

Protein phosphorylation^1^ is a reversible post-translational modification (PTM) in which a phosphate group is covalently attached to specific amino acid residues leading to pronounced changes in protein charge, hydrogen-bonding capacity, and local electrostatics. These physicochemical alterations enable phosphorylation to act as a central regulatory mechanism that modulates protein structure, conformational dynamics, and intermolecular interactions. The phosphorylation state of a protein is dynamically regulated by opposing activities of protein kinases and phosphatases, e.g., protein kinase A^2^ catalyzes phosphate-group attachment, while phosphatases remove these phosphate groups, and the precise coordination of both processes enables accurate spatial and temporal control of protein function. As a result, phosphorylation controls and regulates diverse cellular processes, including signal transduction, enzymatic activity, and protein–protein recognition. Protein tyrosine phosphatases coordinate growth, differentiation, metabolism, cell cycle regulation, and cytoskeletal function by phosphorylating specific tyrosine sites.^3^ Phosphorylation of myosin leads to smooth muscle contraction,^4^ and phosphorylation of light-activated rhodopsin results in arrestin binding, causing receptor desensitization and internalization.^5^ In microorganisms and plants, protein phosphorylation mediated by histidine kinases is essential for regulating various vital processes, primarily via two-component systems and phosphorelays.^6^ Also membrane proteins are phosphorylated, e.g., in G-protein coupled receptors (GPCRs),^7^ phosphorylation promotes arrestin binding and activation.^8^ Thereby, the so-called phosphocode of GPCRs (specific patterns of phosphorylated residues) determines which signaling pathway will occur.^9^ Moreover phosphorylation of GPCRs influences their interactions with the membrane and therefore their activation state.^10^ Another membrane protein family, where phosphorylation plays an important role, are aquaporins.^11–14^ Remarkably, one-third of phosphorylated proteins possess phosphorylation sites inside their hydrophobic core whose exposure can trigger protein degradation.^15^ Misphosphorylation is a prominent disease marker appearing in neurodegenerative diseases, e.g. it increases the toxicity and aggregation of protein fibrils in Alzheimer's disease^16,17^ and can trigger parkin activation and α-synuclein fibrillation in Parkinson’s disease.^18,19^ Furthermore, the phosphorylation of proteins often differs in healthy and cancer cells rendering it an exciting target in personalized cancer diagnostics and therapy.^20–22^ This relevance is underscored by the fact that phosphorylation is observed in approximately one-third of all eukaryotic proteins^23^ and most commonly occurs on the amino acids serine, threonine, and tyrosine (O-phosphorylation)^24^ but is also evident on histidine, arginine, and lysine (N-phosphorylation), as well as aspartic acid (COO phosphorylation). Nonetheless, it has to be noted that not all accessible amino acids are constitutively phosphorylated, rather phosphorylation depends on sequence motifs, protein structure, and cellular context.

As the p*K*_a_ of the phosphate group in phosphorylated amino acids lies close to the physiological pH (5.6 for phosphoserine and 5.9 for phosphothreonine^25^), the dominant charge states are −2 and −1. The introduction of such high local charges to amino acid residues significantly alters their interactions with their surroundings. For example, salt bridge formation plays a crucial role in the secondary structure stabilization and protein–protein interaction^26^ as well as for the solvation, especially on a local level.^19,27^ Moreover, many phosphorylation sites are located in intrinsically disordered regions of proteins, where the reversible PTM phosphorylation can trigger order/disorder transitions.^28,29^ A prominent example protein with extensive disorder and phosphorylation is amyloid-β, the culprit in Alzheimer’s neurodegeneration, making it difficult to characterize it experimentally by methods such as cryogenic transmission electron microscopy (cryo-TEM) or X-ray crystallography.^30,31^

Molecular dynamics (MD) simulations offer the advantage of label-free and therefore perturbation-free structure dynamics at atomic and femtosecond resolution, so that effects such as secondary structure changes in disordered regions due to altered backbone hydrogen bonding can be pinned to specific PTM sites. However, only physically correct force field parameters of PTMs are able to accurately depict the underlying physical behavior. Despite their importance, not all PTMs are covered in all force fields.^32^ Unfortunately, force field parameters of phosphorylated amino acids have been shown to fail at portraying realistic properties and behavior in both Amber and CHARMM force fields: Specifically, in CHARMM36m, salt bridges involving phosphorylation are too strong, in Amber less so but still noticeably.^33,34^ Recently, the parameters of phosphorylated amino acids (Ser, Thr, Tyr, and His) were reparametrized for Amber ff14SB and ff19SB force fields,^35^ yet the quality of these parameters has not been thoroughly evaluated to date, in part due to the limited availability of experimental data suitable for direct comparison, such as osmotic pressure of methylphosphate solutions, high-resolution structural data comparing phosphorylated and nonphosphorylated variants of the same protein, or nuclear magnetic resonance (NMR) data on phosphorylated proteins. For membrane protein simulations, the CHARMM36 force fields are typically the first choice as they contain parameters for numerous membrane compositions of both lipids and sterols. Consequently, parameters for phosphorylated amino acids are essential for these force fields. Currently, the CHARMM36m parameters for phosphorylated serine and threonine originate from parameters for methylphosphate in the CHARMM27 parameter set for nucleic acids.^36^ A previously published study has revealed overaggregation of phosphate and ammonium in solution, resulting in drastically smaller osmotic pressure when compared to the experimental data of a proxy solution of sulfate with ammonium.^37^ The authors of this study suggested the introduction of a pairwise interaction correction, termed NBFIX, between the nitrogen of the ammonium group and the phosphate oxygens. While such pairwise corrections of nonbonded interactions offer a pragmatic route to improving agreement with experimental data, their applicability is fundamentally limited by poor transferability, since each solute–solute or solute–solvent interaction must be treated independently. The NBFIX corrections are often introduced because parametrizing charged molecules is complicated, especially concerning nonbonded interactions, due to the lack of experimental data to compare to, such as heat of vaporization, densities of pure substances, or other properties commonly used for parametrizinguncharged molecules. Here, we use force matching of intermolecular forces between water-solvated molecules from MD and density functional theory (DFT) to improve nonbonded parameters of methylphosphate in different protonation states. Although force matching has been primarily used for obtaining coarse-grained parameters,^38,39^ it has also been successfully applied to all-atom force field parametrization.^40–44^ Here, we evaluate whether force matching of methylphosphate/methylammonium interactions against DFT calculations can improve the agreement between MD simulations and experimental observables for the phosphate group in the CHARMM36m force field. To this end, we have also performed osmotic concentration measurements and studied the dynamics of the phosphorylated dipeptide Lys-pSer by NMR relaxation. Furthermore, we assessed our final parameters in simulations of the anti-sigma factor antagonist SpoIIAA, whose dephosphorylation is involved in the activation of the RNA-polymerase factor σ^F^ required for asymmetric cell division in *Bacillus subtilis*.^45^

## 2 Computational Methods

To briefly summarize our methodology on the reparametrization of methylphosphate in different charge states: Using initial parameters generated by CgenFF,^46^ we performed MD simulations of various methylphosphate systems (see Table S1) in water. From these, common complex conformations, selected by clustering, were geometry optimized by DFT. Using these optimized structures, intermolecular forces were determined at the force field level (MM) and compared to quantum chemical (QC) forces. The force-matching routine then iteratively altered the nonbonded force field parameters in order to obtain a better agreement between the MM and QC forces. As soon as a close match for the intermolecular forces was obtained, the MM parameters were returned, implemented, and tested in MD simulations of osmotic pressure (measured as osmotic concentration), dipeptide dynamics (NMR relaxation times), and proteins.

### 2.1 Obtaining Complex Conformations for DFT Calculations

The small molecules methylammonium (MAM) and methylphosphate (MP), representing the functional groups of the amino acid side-chain analogs of Lys and phosphorylated Ser, respectively, were modeled in Avogadro.^47^ CHARMM topology files were either directly generated in the CHARMM program (Free Version 45b1)^48–51^ or via the CgenFF server.^46^ Notably, methylphosphate was modeled in all three possible protonation states: CH_3_PO_4_H_2_ (MP0), CH_3_PO_4_H^–1^ (MP1), and CH_3_PO_4_^–2^ (MP2). The initial geometries were energy minimized using the steepest descent algorithm for 100 steps, followed by 1000 steps minimization using the Adopted Basis Newton–Raphson (ABNR) algorithm (developed originally by M. Karplus and D.J. States) with a tolerance of 0.01. A periodic, cubic box (4 × 4 × 4 nm) filled with CHARMM-adapted TIP3P^52,53^ water molecules was generated and used for solvating the small molecules. If necessary, a neutralizing amount of Na^+^ or Cl^–^ ions were added. The following solute/ion combinations were placed in the water box: MP0–MP0, MP0–Cl^–^–MAM, MP1–MAM, MP1–Na^+^, MP2–2 MAM, MP2–2 Na^+^ (see Table S1). The entire box was then energy minimized using steepest descent for at least 100 steps and ABNR for at least 300 steps. Next, the system was heated from 110 to 310 K with a heating rate of 0.5 K every 2000 steps using a 1 fs time step, the leapfrog Verlet integrator, particle mesh Ewald (PME)^54^ electrostatics beyond 1.2 nm, and a switching function applied between 1.0 and 1.2 nm. In the simulations, covalent hydrogen bonds were constrained to their equilibrium length using the SHAKE algorithm.^55^ Production simulations were run with the same settings at 310 K and 1 atm for 120 ns in the CHARMM simulation program and its OpenMM plugin to run the simulations on the GPU (omm langevin gamma 0.0 prmc pref 1.0). The coordinates were saved every 1 ps. The amino acid side-chain analogs were then centered in the solvent box and coordinate files, topologies, and trajectories converted to GROMACS-compatible formats using MDAnalysis.^56,57^

In order to obtain a selection of 50 distinct conformations for each system, the trajectories were clustered using gmx cluster with the gromos method,^58^ an RMSD cutoff of 0.05 nm, and least-squares fitting. Only clusters with more than 100 structures were considered. If more than 50 significant clusters were recorded, then we randomly selected 50 of them and extracted their central structures (centromers). For a characterization of the variety of the utilized conformations, see Figure 1.

**Figure 1:**
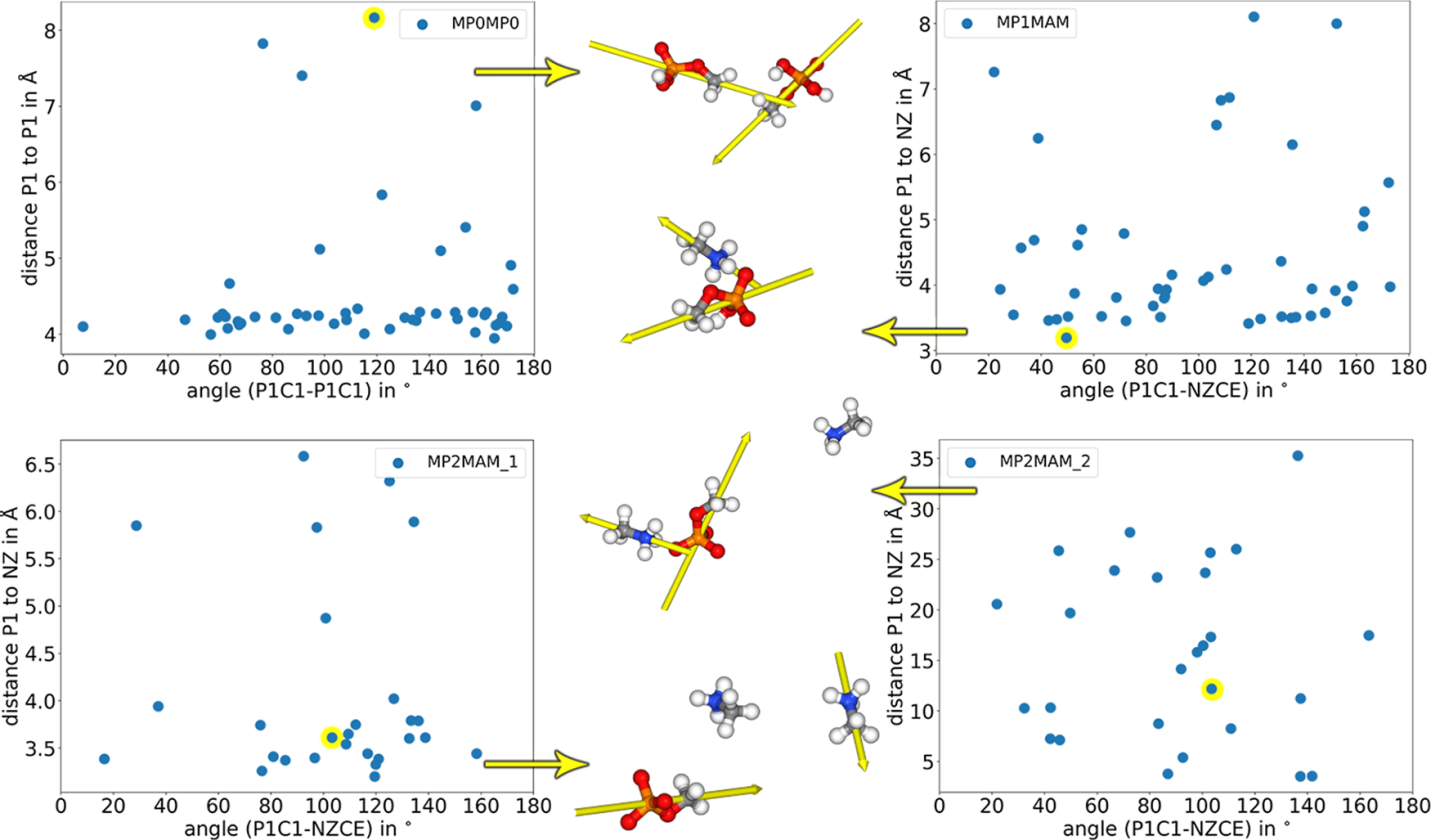
Characterization of different conformations obtained from clustering for simulations containing 2 MP0 molecules (top left), MP1 and MAM (top right), and MP2 with two MAMs (bottom row, left with the first MAM molecule MAM_1 and right with the second MAM molecule MAM_2). A broad spectrum of orientations of the solutes is obtained and later used in the force matching process. In the middle, examples are shown in ball and stick representation. Hydrogens are white, carbon gray, oxygen red, phosphorus orange, and nitrogen blue. The vectors used for the estimation of the relative angles are depicted as yellow arrows through the molecules. The visualized data points are highlighted in yellow.

In the next step, using our own Python code (Box_Slicer, available at https://github.com/CornyC/Box_Slicer), smaller water boxes measuring 1.6 × 1.6 × 1.6 nm were cropped for each of the 50 conformers, containing the amino acid analogs solvated by approximately 113 water molecules. The cropping procedure is visualized in Figure 2.

**Figure 2:**
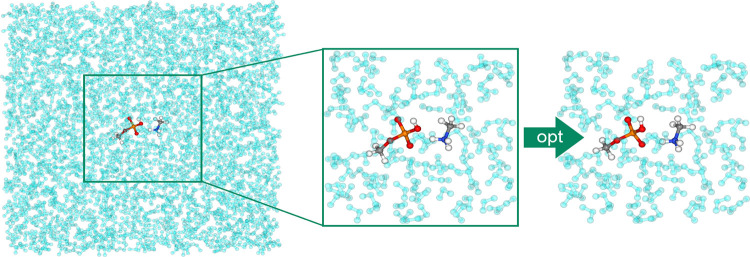
Exemplary frame of one significant conformation indicated by clustering of MP1 and MAM in a 4 × 4 × 4 nm simulation box containing 2003 water molecules. The corresponding cropped 1.6 × 1.6 × 1.6 nm box, containing 112 water molecules, for quantum chemical (QC) calculation is shown in the middle, and the box with the QC-optimized solutes (PBE/TZV2P-MOLOPT-PBE-GTH/DFTD3) is shown on the right.

### 2.2 DFT Calculations

The small water boxes were geometry optimized on the DFT level (TZV2P-MOLOPT-PBE-GTH basis set,^59^ GTH-PBE potentials,^60,61^ PBE functional,^62^ and DFTD3 dispersion correction^63^) using cp2k (versions 8.2 and 9.1)^64^ and the forces on each atom extracted. The input parameters cutoff, rel cutoff, and alpha were converged to 550, 80, and 0.4 for the first system of an uncharged methylphosphate in water, and the thus obtained values were applied to all other systems for consistency. The Gaussian Plane Wave Method^65^ was used with periodic boundary conditions, and water molecules were constrained in place. For the purpose of using net forces between two solutes, e.g., the methylphosphate and its counterion, two water boxes were generated from the geometry-optimized box, with each containing one of the solved solutes only. On these systems, single-point force calculations were performed, and thus obtained forces were subtracted from the forces of the complete system (Figure 3).

**Figure 3:**
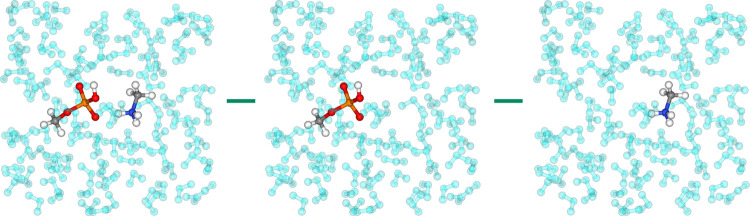
Exemplary visualization of the QC systems used for the net force calculation. Forces obtained at the PBE/TZV2P-MOLOPT-PBE-GTH/DFTD3 level from the boxes with each solute are subtracted from the forces in the entire box to obtain intermolecular forces between the solutes, here MP1 and MAM. In the case of two counterions to MP, both were subtracted at once.

### 2.3 Force Matching and The Phorce Package

Force matching minimizes the difference between forces *f*(*p*_*i*_) acting on atoms *k* in conformations *j* calculated using either quantum chemistry (QC) or classical molecular mechanics (MM), with the latter deriving forces from empirical force fields. The central method is the minimization of the objective function *O*(*p*_*i*_)­

$$O(p_{i})=\sqrt{\frac{1}{3MN}\sum_{k=1}^{N}\sum_{j=1}^{M}|f(p_{i}{)}_{ij}^{\mathrm{QC}}-f(p_{i}{)}_{ij}^{\mathrm{MM}}{|}^{2}}$$ 1

to find an optimal set of parameters *p*_*i*_. *N* describes the number of atoms and *M* the number of conformations. To be able to stress the importance of certain conformations, a weighing term ω_*j*_ can be introduced. To render the residuals dimensionless, the variance Var is typically included:^40^

$$O(p_{i})=\sqrt{\frac{1}{3MN}\sum_{k=1}^{N}\sum_{j=1}^{M}\omega_{j}\frac{|f(p_{i}{)}_{ij}^{\mathrm{QC}}-f(p_{i}{)}_{ij}^{\mathrm{MM}}{|}^{2}}{\mathrm{Var}(f(p_{i}{)}_{ij}^{\mathrm{QC}})}}$$ 2

In our implementation, the variance can be computed either over all atomic forces of each conformation, denoted vpc throughout the manuscript, or over all atomic forces of all conformations (referred to as vc). In order to keep the parameters chemically and physically meaningful, penalty terms can be added to the objective function. See Table S2 for the optimization boundaries implemented in The Phorce and used for our systems.

For each solute system, we loaded the geometry-optimized coordinates of all conformations into The Phorce and used them for computing both quantum and classical forces. The parameter set was exclusively restricted to nonbonded parameters, and only atoms of the phosphate group were reparametrized. A detailed description of the workflow can be found in the Supporting Information.

As CHARMM consistently uses a prespecified charge of 0.09 on aliphatic hydrogens, and the aliphatic carbons typically carry a negative charge completely counteracting the aliphatic hydrogens, the atomic charges *c*_*i*_ of the phosphate group were charge-corrected after force fitting to match the sum of charges of phosphate groups already defined in the CHARMM36m force field^66^ so that

$$\underset{i}{\sum }c_{i,\mathrm{CHARMM}}=\underset{i}{\sum }\left(c_{i,\mathrm{generated}}+\delta_{i}\right)$$ 3

with δ_*i*_ being the charge correction factor. In detail, for MP0, the -PO_4_H_2_ group had a total charge of 0, for MP1, the -PO_4_H^–^ group carried a charge of −1.1, and the -PO_4_^2–^ group had a total charge of −2.

It is important to note that the resulting parameter set is not necessarily unique since multiple nonbonded parameters are optimized simultaneously. Correlations between parameters allow different combinations of nonbonded parameters to reproduce the same reference forces with similar accuracy.

### 2.4 Evaluation against Experimental Observables

To assess the physical behavior and evaluate the quality of the adjusted force field parameters, MD simulations of water-solvated mixtures of MP with MAM as a counterion, an analog of the ammonium side-chain group of Lys, and of Lys–Ser dipeptides in solution were performed, analyzed, and compared to experimentally measured osmotic pressure and NMR relaxation times, respectively. Furthermore, the parameters of phosphorylated serine were assessed regarding physical behavior in simulations of anti-sigma factor antagonist SpoIIAA protein.

#### 2.4.1 Osmotic Pressure Simulations

Osmotic pressure, often measured as osmotic concentration (see eq 6), is a commonly used metric for force field validation or even parametrization.^67^ Here, we followed the strategy of Luo and Roux^68^ and used force walls to perform osmotic pressure MD of MP-MAM solutions. GROMACS^69,70^ (version 2023.2) was selected to run the simulations. Concentration ranges were adapted to the experimental measurements, yielding total concentrations of 0.8, 1.6, 2.4, and 3.2 mol/L (1:1 MP1:MAM) and 0.6, 1.2, 1.8, and 2.4 mol/L (1:2 MP2:MAM). Solute geometries were extracted from the geometry-optimized small water boxes. According to the concentrations, solute molecules were packed into 4 × 4 × 4 nm simulation boxes using gmx insert-molecules, water was added using gmx solvate, and the boxes were energy minimized for 50,000 steps using steepest descent with a tolerance of 100 and a stepsize of 0.01 kJ/mol. An NVT equilibration of 10 ns with a 2 fs time step, LINCS^71^-constrained hydrogens, a grid-based neighbor search updated every 10 steps, the Verlet cutoff scheme,^72^ PME electrostatics^54^ beyond 1.2 nm, and a temperature of 310 K coupled to the system via the v-rescale thermostat^73^ and τ_*T*_ = 0.1 followed. The cubic boxes were placed into 4 × 4 × 10 nm boxes using gmx editconf and the additional compartments filled with water molecules using gmx solvate. The solutes were position restrained (1000 kJ/mol/nm^2^ in all directions) during the following energy minimization and equilibration. The minimization settings were the same as described above and the 10 ns-long NPT equilibration used a 2 fs time step, LINCS-constrained hydrogens, a periodic grid-based neighbor search updated every 10 steps, the Verlet cutoff scheme with rlist = 1.2, a vdW-Potential-switch function between 0.8 and 1.2 nm, PME electrostatics^54^ beyond 1.2 nm, the Potential-shift-Verlet Coulomb modifier, the v-rescale thermostat^73^ with τ_*T*_ = 0.1 and *T* = 310 K, the c-rescale^74^ barostat with semiisotropic pressure coupling, a water compressibility of 4.5^–5^ bar^–1^, a reference pressure of 1 bar, and τ_*p*_ = 5.0. Index groups for all ionic solutes were generated as well as pull groups in order to rededicate GROMACS’ pull code to create semipermeable walls in the boxes at *z* = 3 and *z* = 7 nm. An exemplary jupyter notebook and mdp files can be found at https://github.com/CornyC/Osmotic_pressure_simulations_w_Gromacs_pull_code. The osmotic pressure simulations (NVT ensemble) were run for 30 ns using a 2 fs time step, and the trajectories were saved every 100 steps. Hydrogens were LINCS-constrained, a periodic grid-based neighbor search updated every 10 steps, and the Verlet cutoff scheme with rlist = 1.2, a vdW-Potential-switch function between 0.8 and 1.2 nm, PME electrostatics^54^ beyond 1.2 nm, and the Potential-shift-Verlet Coulomb modifier were used. The Nosé–Hoover thermostat^75^ with *T* = 310 K and τ_*T*_ = 0.1 was applied. The compressibility was set to 4.5^–5^ bar^–1^. Box pressure coupling was semiisotropic. Each pull group had flat-bottom potentials with a force constant of 1000 kJ/mol/nm^2^ assigned at both walls in the *z*-direction. The pulling geometry was set to direction-periodic. Prior to analysis, molecules crossing periodic boundaries were made whole using gmx trjconv with the flag -pbc mol. The strength of the flat-bottom potential was validated by visual inspection of the trajectories in nglview,^76^ which revealed that the solutes were indeed not able to fully pass through the simulated semipermeable membrane. The osmotic pressure was calculated from the simulations according to Luo and Roux^68^ by first extracting the *z*-coordinates (in nm) of the ionic solutes *i* whose center of mass (COM) had passed the virtual membrane and calculating the absolute distance between their COM and the *z*-coordinate of the wall *z*_wall_, summing over them and the number of frames in the trajectory, *N*, dividing by *N*, and multiplying by the force constant *k* = 1000 kJ/mol/nm^2^ to obtain the mean force ⟨*F*_wall_⟩:

$$\left\langle F_{\mathrm{wall}}\right\rangle =k\cdot \frac{1}{N}\cdot \underset{N}{\sum }\underset{i}{\sum }\left|z_{i}-z_{\mathrm{wall}}\right|$$ 4

with |*z*_*i*_| > |*z*_wall_|. From the mean force, the osmotic pressure Π is obtained by division by the membrane area *A*, in our case 2 × 4 × 4 = 32 nm^2^:

$$\Pi =\frac{\left\langle F_{\mathrm{wall}}\right\rangle }{A}$$ 5

The osmotic pressure is proportional to the osmotic concentration *c*_osm_:

$$\Pi =R\cdot T\cdot c_{\mathrm{osm}}$$ 6

with *R* being the gas constant and *T* the temperature. Therefore, the osmotic concentration is temperature independent.

#### 2.4.2 Calculating NMR Relaxation Times

Solution nuclear magnetic resonance (NMR) can be used to study the rotational dynamics of solute molecules by measuring the longitudinal relaxation time *T*_1_ and the transverse or spin–spin relaxation time *T*_2_. These can also be calculated from MD simulations.^77^ As force field parameters are known to strongly influence peptide rotational dynamics,^77,78^ we here quantified the influence of the phosphate nonbonded parameters on NMR relaxation times by calculating *T*_1_ and *T*_2_ from MD trajectories and evaluating them against experiments. Specifically, we have modeled the dipeptide KS and two differently charged states of the dipeptide KpS. The latter dipeptide consists of a charged lysine and a phosphoserine that is either fully protonated or singly protonated. To match the experimentally investigated dipeptides, the dipeptides were capped at the N-terminus (Lys) with an acetyl group in Avogadro^47^ and at the C-terminus (phosphoserine) by addition of an amine group in GROMACS using gmx pdb2gmx (see Figure 4).

**Figure 4:**
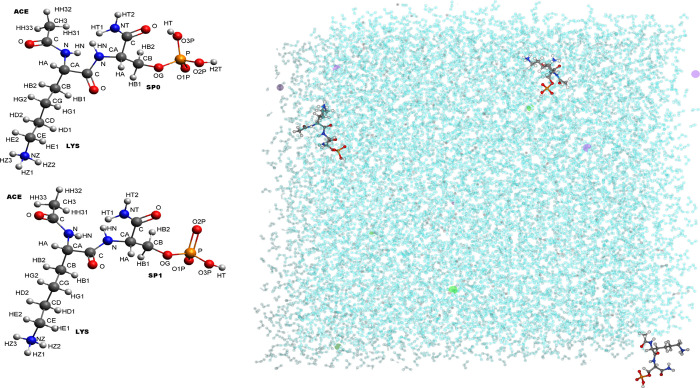
Top left: Ac–K^+^–pS^0^–NH_2_ at pH = 2, i.e., where both lysine (LYS) and phosphoserine (SP0) are fully protonated. Bottom left: Ac–K^+^–pS^–^–NH_2_ at pH = 4.5, i.e., where lysine (LYS) is fully protonated and phosphoserine (SP1) singly protonated, i.e., carrying the charge −1. Right: Cubic simulation box including 3 dipeptides Ac–K^+^–pS^–^–NH_2_, 7 Na^+^ (purple dots) and 7 Cl^–^ ions (green dots) and 8789 water molecules (cyan) used for the estimation of the NMR relaxation times from MD simulations.

Three copies of each dipeptide were put into cubic boxes with an edge length of 6.47 nm, using gmx insert-molecules, and water was added using gmx solvate. The tool gmx genion was used to add 4 Na^+^ (uncharged phosphate group and KS)/7 Na^+^ (−1 charged phosphate group) and 7 Cl^–^, followed by a steepest descent minimization for 1000 steps and a tolerance of 10 kJ/mol. The boxes were then equilibrated for 10 ns with a step size of 1 fs. The Berendsen thermostat^79^ with τ_*T*_ = 0.5 ps and *T* = 298 K controlled the temperature, and the Berendsen barostat^79^ was isotropically coupled to the system with τ_*p*_ = 5.0 ps, *p* = 1 bar, and a compressibility of 4.5 · 10^–5^ bar^–1^. Nonbonded interactions were treated with a periodic neighbor list updated every 10 steps, the Verlet cutoff scheme, and a potential-switching function between 0.8 and 1.2 nm. Electrostatics were modeled by PME^54^ beyond 1.2 nm with the Potential-shift-Verlet Coulomb modifier. Production simulations were run for 1 μs with a time step of 2 fs, the Nosé–Hoover thermostat, and the c-rescale barostat.^74^ Molecules split over periodic boundary conditions were made whole with gmx trjconv and -pbc mol. Index files for CH_*x*_ and NH_*x*_ groups were generated and water molecules removed from the trajectories before running gmx rotacf to compute rotational autocorrelation functions. The Redfield equations^80,81^ link the experimentally observable relaxation times to the rotational correlation functions obtained from MD.^77^ The Python code can be found in our DaRUS repository at 10.18419/darus-5682 in relaxation_times_from_MD.zip.

### 2.5 Protein Simulations

The crystal structures of the anti-sigma factor antagonist SpoIIAA protein 1H4Y^45^ and 1H4X^45^ were obtained from the protein databank (RCSB) and adapted to the CHARMM36m force field using CHARMM-GUI’s pdbreader.^82–85^ Thus, the obtained pdb files were processed by GROMACS’ pdb2gmx to generate topology files with NH_3_^+^ and COO^–^ termini and standard protonation states of all residues (except pS57, which was generated either in a single protonated or fully deprotonated state). All histidines carried the hydrogen on N_δ_. Next, gmx editconf and gmx solvate were used to solvate the proteins in CHARMM TIP3P water in cubic boxes measuring 6.3 × 6.3 × 6.3 nm. Approximately 20 Na^+^ and Cl^–^ (0.15 M) were added to the solution in a neutralizing manner. The systems were energy minimized three times, once in between with the protein backbone restrained, all times using steepest descent, once for 1000 steps, and twice for 500,000. During the first equilibration phase (10 ns, NPT ensemble), the protein backbone was restrained. Thereby, the hydrogens were LINCS^71^-constrained, a grid-based neighbor search was updated every 10 steps, and the Verlet cutoff scheme was used.^72^ PME^54^ was used to treat electrostatics beyond 1.2 nm with the Potential-shift-Verlet Coulomb modifier. The Potential-switch vdW modifier was applied in between 0.8 and 1.2 nm. The system was coupled to the temperature bath of 310 K via the v-rescale thermostat^73^ and τ_*T*_ = 0.1. The pressure was coupled by the c-rescale barostat^74^ to 1 bar using the compressibility of 4.5 × 10^–5^ bar^–1^. Next, an unrestrained equilibration was performed for 30 ns with a 2 fs time step, and the same settings for treating the nonbonded interactions and the same temperature and pressure as before were used, followed by the Berendsen thermostat and barostat^79^ with isotropic pressure coupling, τ_*T*_ = 0.5 and τ_*p*_ = 5.0, and LINCS-restrained^71^ hydrogens. The production simulations were run for 1 μs using a 2 fs time step at 310 K and 1 bar and a compressibility of 4.5 × 10^–5^ bar^–1^. The Nosé–Hoover thermostat^75^ and the c-rescale barostat^74^ with isotropic pressure coupling, τ_*T*_ = 0.5 and τ_*p*_ = 5.0, and LINCS-constrained^71^ hydrogens were used. A grid-based neighbor search was updated every 10 steps, and the Verlet cutoff scheme was used^72^ with PME^54^ to treat electrostatics beyond 1.2 nm with the Potential-shift-Verlet Coulomb modifier. The Potential-switch vdW modifier was applied in between 0.8 and 1.2 nm. Root mean square deviation (RMSD) of C_α_ atoms after a fit on the C_α_ atoms of the corresponding crystal structure was conducted in MDAnalysis^57^ exactly like the residue-wise root-mean-square fluctuation (RMSF) analyses performed using C_α_ atoms. DSSP secondary structure analysis^86^ was done using GROMACS 2021 do_dssp -ver 2. Simulation files can be found in our DaRUS repository at 10.18419/darus-5682.

## 3 Experimental Methods

### 3.1 Osmotic Concentration Measurements

In osmotic concentration measurements, methylphosphonic acid was used as a proxy for methylphosphate, as the latter cannot be obtained in pure form due to its intrinsic instability of the C–O–P linkage in small molecules and tendency to exist as an equilibrium mixture of mono- and dimethylphosphate. The commercial availability of a mixture of methylphosphate and dimethylphosphate only made us choose a methylphosphonic acid:methylammonium mixture. Notably, osmotic concentrations of Ca^2+^:dimethylphosphate^–1^ mixtures have been estimated before,^67^ yet dimethylphosphate is not a chemically suitable analog of phosphorylation.

Methylphosphonic acid (MPA, 98%) and methylamine (MAM, 40 wt % in H_2_O), both from SigmaAldrich, were used to prepare a 4 M solution each. For this purpose, 0.768 g of MPA and 682 μL of the 40 wt % MAM solutions were filled up to 2 mL with distilled H_2_O. These 4 M MPA and 4 M MAM stocks were used to prepare two mixing ratios of 1:1 MPA-MAM and 1:2 MPA-MAM with a total molar concentration of 4 M. For the 1:1 MPA-MAM mixture, 500 μL of the 4 M MPA stock and 500 μL of the 4 M MAM stock were mixed, resulting in 2 M MPA and 2 M MA. For the 1:2 MPA-MAM mixture, 350 μL of 4 M MPA stock and 700 μL of 4 M MAM stock were mixed, resulting in 1.33 M MPA and 2.67 M MAM. For both mixtures, a linear dilution series was prepared. The prepared mixtures with defined molar concentration (M) were then used to experimentally determine the corresponding osmotic concentration (*c*_osm_). Three independent measurements were taken. Therefore, a freezing point osmometer (Knauer K-7400S Semi-Micro Osmometer) was used, which could measure total molar concentrations up to around 2 M. For the 1:1 MPA-MAM mixture, data points between 0 and 1.89 M and for the 1:2 MPA-MAM mixture data points between 0 and 2.11 M could be obtained. The pH of the solutions (provided in detail in Supplementary Table S5) were determined using a pH Meter (Seven Compact S210) and Electrode (InLab Expert Pro-ISM) from Mettler Toledo. Thereby, 1 M solutions were chosen as representative midrange concentrations of the measurement series. To assess the stability of the pH, three additional dilutions (0.5, 0.25, and 0.125 M) of each solution were additionally measured. The resulting pH amounted to 3.23–3.36 for MPA-MAM 1:1 solution and 7.82–7.99 for MPA-MAM 1:2 solution.

To test the reliability of the osmotic concentration values, a dilution series of NaCl was measured as a standard and compared with values from the literature.^87^ To this end, a 4 M NaCl solution was prepared by dissolving 468 mg of NaCl in distilled H_2_O and filling the solution up to 2 mL.

### 3.2 NMR Measurements

#### 3.2.1 Sample Preparation

The unlabeled lysine–serine (KS) dipeptide and its phosphorylated analog KpS, both N-terminally acetylated and C-terminally amidated, were obtained from Peptide Protein Research Ltd. (Fareham, United Kingdom) and Biomatik Corporation (Kitchener, Canada), respectively. The capping groups neutralize the effects of terminal charges and introduce a third peptide bond as well as an additional C_α_, thereby mimicking the intrachain chemical environment of a polypeptide. Lyophilized samples were dissolved in 90% H_2_O, 10% D_2_O, and 15 μM sodium trimethylsilylpropanesulfonate (DSS) to a final concentration of approximately 5 mg/mL. NMR spectra were acquired in 5 mm Bruker (Billerica, MA, United States) tubes with a sample volume of 600 μL. Measurements for KpS were performed at an initial pH of 1.65 and subsequently at pH 2.00 and 4.50, while the stability of the pH was guaranteed due to the high KpS concentration and its low self-buffering; pH values were adjusted by titration with NaOH and verified using a pH meter. Measuring Ac–K^+^pS^–2^–NH_2_ would require a pH > 10, at which the chemical exchange of protons becomes too fast to detect an NMR signal. The pH of KS solution was not adjusted. The lysine side-chain ϵ-amino group, with a p*K*_a_ of approximately 10.5, is therefore protonated under the near-neutral conditions expected for an unbuffered aqueous KS solution.

#### 3.2.2 Acquisition and Spectra Processing

NMR data for both KS and KpS were acquired using two Bruker BioSpin spectrometers: an Ascend 600 MHz equipped with a QCI-P CryoProbe and an Ascend 850 MHz with a TCI CryoProbe. Both instruments were operated with an AVANCE NEO console, and sample handling was fully automated via a SampleJet sample changer. All samples were stored at 4 °C and equilibrated to 298 K for at least 2 min prior to data acquisition.

The chemical shift assignments for KS have been reported previously,^88^ and those for KpS were acquired using the same procedure at 600 MHz, including the corresponding ^31^P resonances. *T*_1_ and *T*_2_ relaxation measurements were performed at 298 K for both KS and KpS at 600 and 850 MHz for all NMR-accessible ^13^C and ^15^N nuclei, at natural abundance. The relaxation times of ^15^N were measured using 1D versions of standard HSQC-based relaxation experiments,^81,89,90^ while ^13^C relaxation was observed directly. Details of all NMR experiments and the associated acquisition settings are given in Table 1.

**Table 1:** Overview of Selected Acquisition Parameters for NMR Experiments: Number of Scans (NS), Number of Time Domain Points in the F1 Dimensions (1TD), Spectral Width (SW), and Spectral Center (O1P)^a^ ^b^ ^c^ ^d^

**NMR experiment**	**pulse sequence**	**number of scans**	**1TD**(F1) points	spectral width/center [ppm]	**remark**
1D ^31^P	zgpg30	32	65 536	395.9/–50.0	^31^P assignment
1D ^15^N T_1_ NH-optimized	hsqct1etf3gpsi	32	4 096	16.0/4.7	cnst11 = 4, cnst12 = 4, delay list 1
1D ^15^N T_1_ NH_2_-optimized	hsqct1etf3gpsi	32	4 096	16.0/4.7	cnst11 = 8, cnst12 = 8, delay list 1
1D ^15^N T_2_ NH-optimized	hsqct2etf3gpsi	64	4 096	16.0/4.7	cnst11 = 4, cnst12 = 4, delay list 1
1D ^15^N T_2_ NH_2_-optimized	hsqct2etf3gpsi	128	4 096	16.0/4.7	cnst11 = 8, cnst12 = 8, delay list 1
1D ^13^C T_1_	t1irigsp.cga	32	65 536	236.7/100.0	delay list 2
1D ^13^C T_2_	cpmgigsp.cga	128	65 536	236.7/100.0	delay list 3

a For all *T*_1_ and *T*_2_ relaxation experiments, a recycling delay of 15 s between scans was applied. All pulse sequences were included in TopSpin (Version 4.1.4, Bruker BioSpin).

b Delay list 1: 0.55, 1, 1.75, 3.2, 5.5, 10, and 15 s.

c Delay list 2: 0.175, 0.32, 0.55, 1, 1.75, 3.2, 5.5, and 10 s.

d Delay list 3: 0.176, 0.24, 0.36, 0.6, 0.96, 1.44, 2.04, and 2.78 s.

All spectra were processed in Bruker’s TopSpin (version 4.1.4) using standard processing routines: The time-domain data were zero-filled to 131 072 (128k) points for ^13^C and 8 192 (8k) points for ^1^H (^15^N relaxation) prior to Fourier transformation, and resulting spectra were referenced to DSS. For all 1D relaxation time data sets, an exponential multiplication window function was applied with line-broadening factors of 1 Hz for ^13^C and 15 Hz for ^1^H (^15^N relaxation) to obtain Lorentzian line shapes. For each *T*_1_ or *T*_2_ data set, the spectrum with the shortest delay was used for automatic phase correction, and the resulting parameters were applied to all other spectra of the set, followed by individual baseline correction of each spectrum.

#### 3.2.3 Determination of Relaxation Times

To extract the relaxation times *T*_1_ and *T*_2_, the processed spectra were analyzed in Python by fitting the individual resonances to a Lorentzian line shape *L*(*p*), in which *p* represents the chemical shift (eq 7). Peak fitting was performed using the LMFIT package^91^ in combination with the Levenberg–Marquardt optimization algorithm.^92,93^ Approximate peak positions were known from the assignments and used as initial values. During fitting, the exact peak center *p*_0_ was refined together with the peak’s half width at half-maximum (HWHM) γ and the integrated peak intensity *I*—with *I* being a measure corresponding to the total area under the Lorentzian peak.

$$L\left(p\right)=\frac{I}{\gamma \cdot \pi }\cdot {\left(1+{\left(\frac{p-p_{0}}{\gamma }\right)}^{2}\right)}^{-1}$$ 7

The relaxation times *T*_1_ and *T*_2_ were calculated by fitting the decay of *I* as a function of the respective delay times to a single-exponential, using the LMFIT package together with the Levenberg–Marquardt minimization algorithm, while the associated experimental errors were estimated from the uncertainties of the fit. Relaxation times extracted from NH- and NH_2_-optimized experiments were subsequently combined using weighted averaging, with weights (*w*) being calculated from the individual errors (ϵ) and the number of data points (*n*) included in each exponential fit:

$$w=\frac{n-2}{\epsilon }$$ 8

and the corresponding combined error (ϵ_tot_) following

$$\epsilon_{tot}=\sqrt{\frac{{\left(w_{1}\epsilon_{1}\right)}^{2}+{\left(w_{2}\epsilon_{2}\right)}^{2}}{{\left(w_{1}+w_{2}\right)}^{2}}}$$ 9

## 4 Results and Discussion

Using our Python package The Phorce, new CHARMM36m-compatible nonbonded parameters were generated based on our QM data sets for MP0–MP0 in water, MP1–Na^+^, MP1–MAM, and MP2–2MAM (Figure 5). The highly uneven potential surface, restrictions imposed by parameter constraints, and the wrapped objective function, which couples multiple code and algorithmic instances across different levels, pose significant challenges for force-matching optimization, making the resulting objective function not differentiable. Therefore, SciPy’s local Nelder–Mead optimizer was selected as it does not require gradient information and yielded the most efficient convergence. An overview of the generated parameters, together with the number of iterations required for convergence, is provided in Table S4. The full-precision values can also be found in the files in the DaRUS repository.

**Figure 5:**
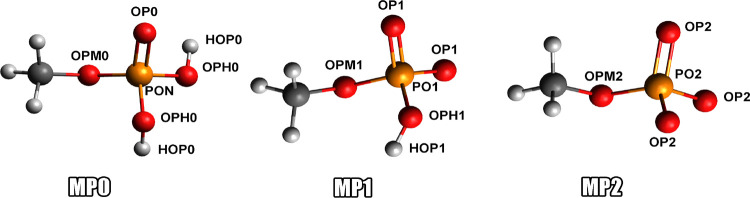
Mapping of the here-defined atom types of MP0, MP1, and MP2 representing the amino acid side-chain analogs of phosphorylated serine residues SP0, SP1, and SP2, respectively. Hydrogens are shown as white spheres, carbons as dark gray spheres, oxygens as red spheres, and phosphorus as orange spheres.

All our generated parameters were successfully used in MD simulations of test systems and did not cause simulation instabilities or unphysical behavior upon visual inspection. To probe their applicability and physical behavior more closely, we compare them in the next sections to experimentally measured observables, namely, osmotic concentration and spin–lattice (*T*_1_) and spin–spin (*T*_2_) relaxation times. For the purpose of osmotic concentration, MP1 or MP2 with neutralizing amounts of MAM in aqueous solution was simulated (see Figure 6) and compared against measured solutions of MPA and MAM in water. For the determination of relaxation times, the small dipeptides (KS and KpS) in water were simulated and also measured experimentally using NMR, with KpS studied in two different protonation states (see Figure 4). Afterward, MD simulations of the anti-sigma factor antagonist SpoIIAA protein in nonphosphorylated and phosphorylated states were performed and analyzed.

**Figure 6:**
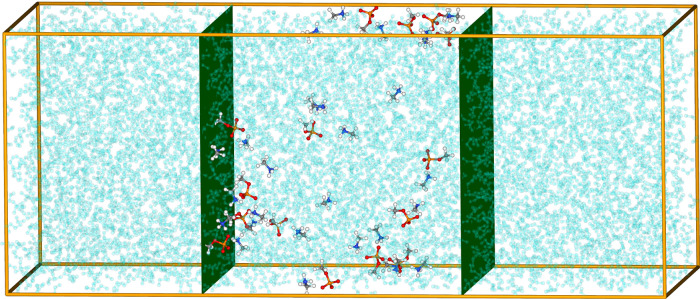
Simulation setup for osmotic pressure simulations exemplarily shown for the MP2–MAM 1:2 solution in a total concentration of 1.2 mol/L using the MP2–2MAM vc parameters and the CHARMM TIP3P water model. The solutes are shown in ball + stick representation, hydrogen atoms are colored white, oxygens red, phosphorus atoms orange, and nitrogens blue. The two force boundaries which are acting only on solutes, i.e., on MP2 and MAM, are visualized as green surfaces. The *z*-axis is located in the left–right direction.

### 4.1 Osmotic Concentrations of Methylphosphate/Methylammonium Solutions

#### 4.1.1 The Role of Water Models in Osmotic Pressure

Ion solvation in water is a highly complex process that affects not only the dynamics and interactions of the ions themselves but also has essential effects on all other molecules in solution, with a particularly strong influence on the stability, dynamics, and interactions of proteins.^94,95^ Therefore, we began our study of osmotic concentration simulations by analyzing the effect of different water models on the osmotic concentration of MP1–MAM and MP2–2MAM using standard CHARMM36m parameters. The results using CHARMM TIP3P,^52,53^ TIP4P,^96^ TIP4P/ε,^97,98^ and OPC^99^ are shown in Figures 7 and 8, left. Overall, the water models altered the osmotic concentration maximally by −21% (TIP4P, 0.6 mol/L, MP2) and 173% (OPC, 2.4 mol/L, MP2), compared to CHARMM TIP3P. Thereby, the slower diffusing TIP4P gives overall slightly smaller osmotic concentration values and is thus in worse agreement with the experiment, while the plug-and-play OPC,^99^ which has been recently selected as a preferred water model for Amber19sb,^100^ slightly improves the results. It is important to note here that, to the best of our knowledge, OPC has not been systematically tested and implemented for protein simulations with CHARMM36m yet, although there are first examples in the literature on its usage in comparison of MD and NMR data.^101^ TIP4P/ε, a water model whose pure properties like self-diffusion^102^ and dielectric constant are in great agreement with experiments,^97,98^ and which was thus hypothesized by us to potentially better solvate charged species, also leads to an improvement compared to CHARMM TIP3P and TIP4P and nearly reaches the OPC values (see Figures 7 and 8). Thus, water models influence ion–ion interactions and modify their osmotic concentration. Yet, the deviation of the osmotic concentration of MP1–MAM and MP2–MAM solutions from the experimental values resulting from too strong attractive forces between the ions cannot be corrected for by incorporating any of the water models tested here. Therefore, in all our following adjustments, the standard CHARMM TIP3P water was consistently used.

**Figure 7:**
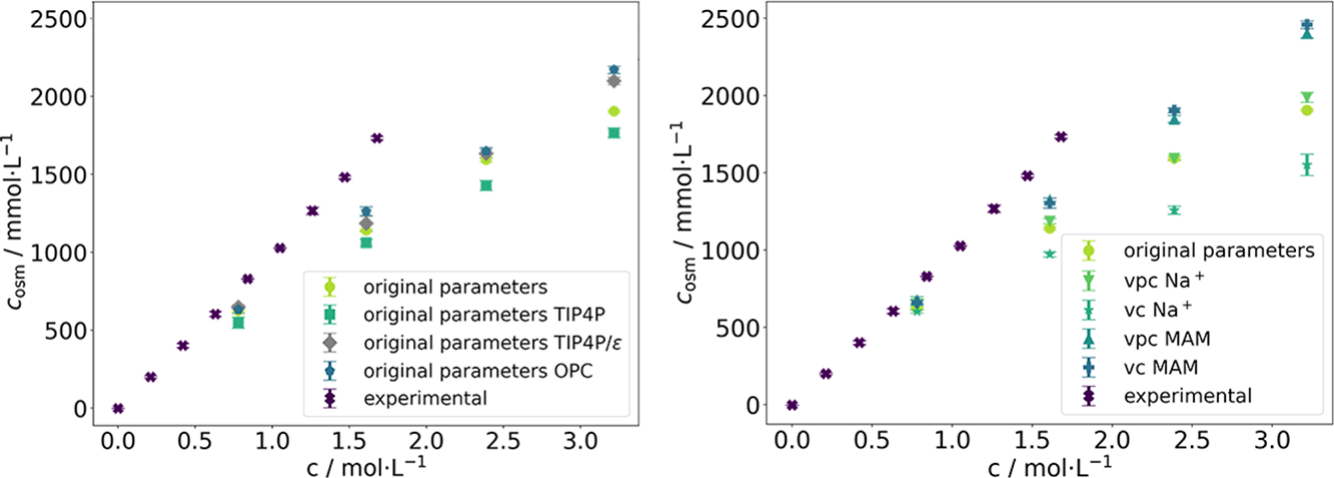
Comparison of the osmotic concentration of MP1–MAM solutions in simulations to experimental osmotic concentrations of MPA–MAM 1:1 solutions. Osmotic concentrations are plotted against total molar concentrations of both electrolytes. Left, the effect of different water models, i.e., standard CHARMM TIP3P, TIP4P, TIP4P/ε, and OPC on the osmotic activity using the original CHARMM36m force field parameters (original parameters). Right, comparison of the here developed parameters for MP1 (vc and vpc stand for variance over all conformations and over each conformation, respectively, Na^+^ or MAM indicates the counterion used in the force-matching procedure) to the original CHARMM36m parameters (all simulated together with the CHARMM TIP3P water model) and to the experimentally obtained values. The error bars show the standard error of the mean over 3 independent replicas.

**Figure 8:**
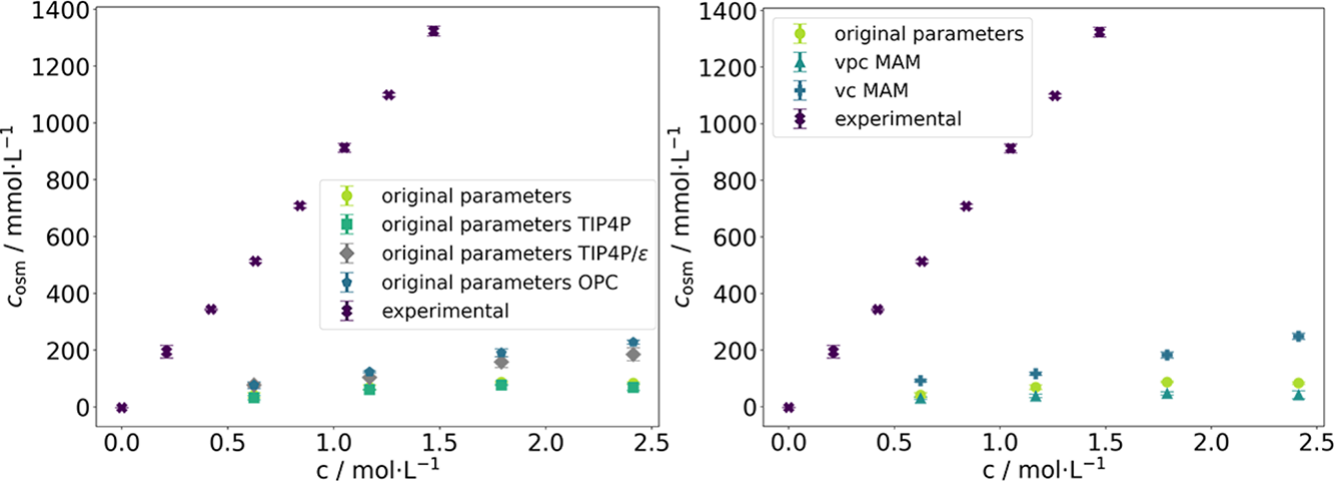
Comparison of the osmotic concentration of a MP2–2MAM solution in simulations compared to experimental values of a MPA-MAM 1:2 solution. Osmotic concentrations are plotted against total molar concentrations of both electrolytes. Left, the effect of different water models, i.e., standard CHARMM TIP3P, TIP4P, TIP4P/ε, and OPC on the osmotic concentration using the original CHARMM36m force field parameters (original parameters). Right, comparison of the here developed parameters for MP2 to the original CHARMM36m parameters (all simulated in the CHARMM TIP3P water model) and to the experimentally obtained values. The error bars show standard error of the mean over 3 independent replicas.

In the next step, we were interested whether our force matching reparametrization of MP1’s and MP2’s nonbonded parameters yields a better agreement of osmotic concentrations between simulation and experiments. Notably, the parameters of MAM were not modified as their change would alter interactions of lysine side chains with all other amino acids and molecules and thus potentially compromise the compatibility with the CHARMM36m force field.

#### 4.1.2 Performance of MP1 Parameters

For MP1, we generated four sets of our MP1 parameters by force matching and compared them against the original CHARMM36m parameters and against experimentally measured values (see Figure 7, right). Except for the parameter set obtained from the vc MP1–Na^+^ interactions, which yielded osmotic concentrations up to 21% (at 2.4 mol/L) lower than the original CHARMM parameters, our parameters provide osmotic concentrations closer to the experimental values. The vpc MP1–Na^+^ data set increases the osmotic concentration only by up to 5% (at 3.2 mol/L), while the data sets parametrized against the interaction with MAM boost the osmotic concentration by up to 26% (vpc MAM, 3.2 mol/L) and up to 29% (vc MAM, 3.2 mol/L), with the vc MP1–MAM being the generally best-performing data set providing osmotic concentrations in a close agreement with the experimental values. It is interesting to note that the vc and vpc charge parameters for phosphorus in the MP1–MAM system are relatively similar (*P* charge of vc: 1.64 and vpc: 1.53, respectively), whereas substantially larger values are obtained when fitting to the MP1–Na^+^ data sets (*P* charge of vc: 3.26 and vpc: 2.75, respectively). This difference suggests that the interactions observed in MP1–Na^+^ solutions cannot be fully compensated by adjusting the parameters of methylphosphate alone, but are likely influenced by the specific nonbonded interactions of the Na^+^ ion. As the primary goal of this work is to obtain reliable parameters for methylphosphate in combination with methylammonium as a model for phosphorylated amino acids, only MP–MAM systems were considered.

#### 4.1.3 Performance of MP2 Parameters

For MP2 carrying a total charge of −2, the osmotic concentration from all simulations is far from the experimental results (Figure 8), even though the MP2–2MAM vc data set again exhibits an improvement over the original parameters. The reason for the drastically underestimated osmotic concentrations of the MP2–2MAM solutions is clotting of the solutes (see Supplementary Figure S1 for visualization of a behavior of a 1.2 mol/L MP2–MAM 1:2 solution described by the original CHARMM36m parameters and the CHARMM TIP3P water model). The inability of standard CHARMM parameters of anions like acetate, sulfate, and dimethylphosphate with ammonium to reproduce osmotic pressure is well documented.^37^ As mentioned in the Introduction already, one solution proposed in the literature is to introduce pair-specific corrections to the nonbonded interactions (NBFIX) between selected ions, for example, phosphate and ammonium^37^ or between Ca^2+^ and dimethylphosphate^1–^.^67^ While this approach is pragmatic and can yield a high accuracy of the osmotic pressure for a given solute pair, it lacks generality. Each interacting pair must be independently reparametrized, requiring extensive experimental osmotic pressure data for solute mixtures and undermining the transferability that is central to force field development. To learn the effect nonbonded parameters exert on osmotic concentration, we manually varied the nonbonded parameters of the phosphate group in MP2. These variations are discussed in the Supporting Information.

#### 4.1.4 Obtaining Final MP2 Parameters and Testing Their Transferability

In general, ions and especially bivalent ions and their solvation are difficult to simulate by classical fixed-charge force fields, because the missing electronic polarization leads to their overbinding. One possible solution is the above-discussed introduction of pairwise NBFIX parameters, forcing two ions further apart.^37,67^ Alternatively, to enforce proper hydration, Yoo et al. suggested a custom model of hydrated calcium ions,^103^ where six water molecules are explicitly bonded to the calcium dication. Despite the ability of this model to properly describe the fully hydrated Ca^2+^, this model will intrinsically fail in the description of direct ion–ion interactions. Another possibility, mainly investigated in the group of Pavel Jungwirth, is to scale down the charges of all ions reflecting their effective charge in a hydrated state. This approach is also known under the term electronic continuum correction^104^ and is able to diminish the overbinding of ions compared to the uncorrected force field, e.g., Na^+^ and Ca^2+^ binding to a POPC bilayer,^105^ various salt ions binding to proteins,^106^ and Na^+^ and K^+^ binding to polyelectrolytes.^107^ Thus, to avoid unphysical changes to the RDF caused by altered Lennard-Jones parameters (see Supplementary Figure S4) and being inspired by Fan et al.,^104^ we decided to scale the total charge of MP2 down to −1, which would also be closer to the charge value obtained from The Phorce (in detail, the phosphate group of MP2 carried a charge of −0.85 in both MP2–2MAM parameter sets) and investigate its influence on the osmotic concentration. As a basis, the MP2–2MAM vc parameter set was used, and the charges were adapted to OPM2: −0.68, PO2: 1.75, OP2: −0.69. As it can be seen in Figure 9, reducing the charge of MP2 to −1 yields osmotic concentrations perfectly matching the experimental ones. The reduced charge influences the RDFs, indicating the hydration shells contain slightly less waters and are minimally shifted to longer distances, yet the basic structure is not disturbed as in the NBFIX data set and the second hydration shell is exactly in the middle of the 4 and 5 Å range from the higher level theory (ab initio quantum mechanical charge field molecular dynamics^108^ and Born–Oppenheimer MD simulation^109^) (see Supplementary Figure S6). As the charge scaling drastically improved the osmotic concentration, we investigated whether scaling the charge of the original CHARMM36m parameters for MP2 to −1 alone improves the osmotic concentration for the original parameters. The results, plotted in Supplementary Figure S7, indeed show a drastic improvement. Additionally, to examine whether the relatively high positive charge of the phosphorus atom in our final parameters influences interactions with MAM, the charge was partially shifted to the neighboring carbon, reducing the phosphorus charge from +1.75 to +1.1, corresponding to the original CHARMM36m parameters, and increasing the carbon’s charge from −0.27 to +0.38. Chemically, the carbon atom does not interact with water or ions as it is sterically hindered by the surrounding CH_3_ hydrogens, which carry standard CHARMM36m parameters for aliphatic hydrogens. Indeed, the osmotic concentration using this charge-shift was only marginally different from that of our final parameters (see Supplementary Figure S7, right). Notably, this result legitimates our exclusion of the methyl carbon atom from parameter fitting in our force-matching procedure. In the same plot, the results of another test can be found, namely, osmotic concentrations of MP2–2MAM solutions described by the Amber19sb force field and OPC water. Amber19sb gives intrinsically higher osmotic concentrations than the original CHARMM36m parameters with MP2 carrying a charge of −2, yet still underestimates the experimental values, yielding results in between the original CHARMM36m and our final parametrization.

**Figure 9:**
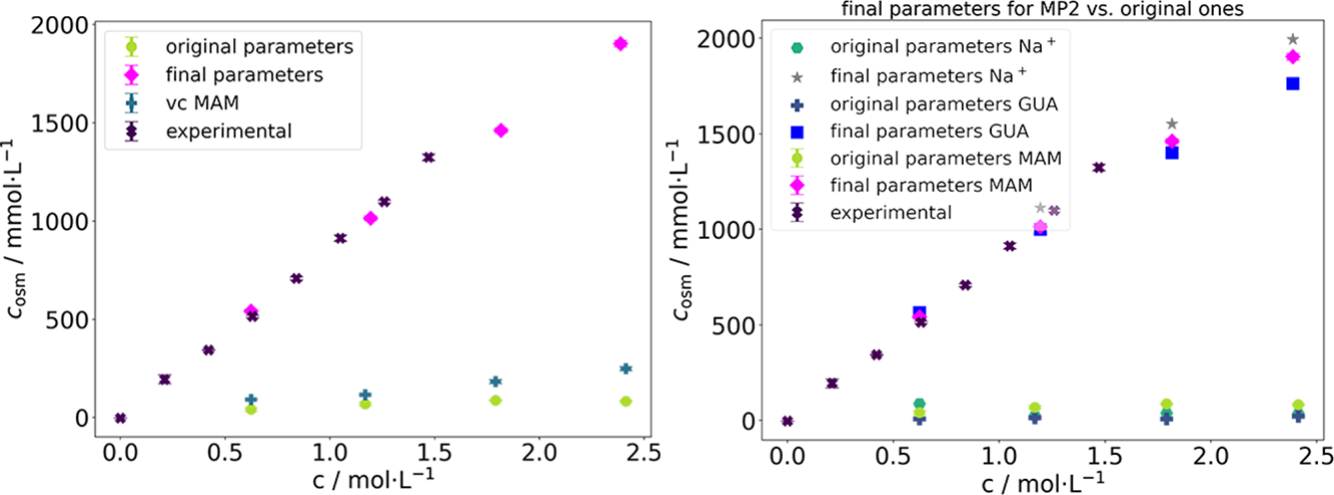
Left: Comparison of osmotic concentrations from simulation of MP2^–1^-1MAM (vc MP2^–1^-1MAM, corresponding to our final parameters) to the MP2–2MAM simulations using original CHARMM parameters, or our MP2–2MAM vc parameters (vc MAM), and experimental values of MPA-MAM 1:2 solutions. All simulations are performed in CHARMM TIP3P water. Osmotic concentrations are plotted against total molar concentrations of both electrolytes. The vc MP2^–1^-1MAM parameter set is based on the Lennard-Jones parameters from the MP2–2MAM “vc MAM” data set while the charges were modified to OPM2: −0.68, PO2: 1.75, OP2: −0.69, yielding a total charge of −1 for MP2 in the simulation. Right: Comparison of osmotic concentrations from simulation using the MP2^–1^-1MAM (vc MP2^–1^-1MAM) parameters for methylphosphate, denoted as final parameters, together with different counterions (Na^+^ and guanidinium) described by the original CHARMM36m parameters.

Finally, to assess the transferability of the parameters, we evaluated the osmotic concentration of MP2–Na^+^ and MP2-guanidium solutions using the methylphosphate parameters derived from the vc MP2^–1^-1MAM data set (final parameters) and counterions carrying standard CHARMM36m parameters. These tests allow us to determine whether parameters optimized for the MP2–MAM system also provide a consistent description of solutions containing a different counterions. Experimentally, NaCl, MPA-MAM, and MPA-2MAM solutions exhibit very similar osmotic concentrations when plotted against the total ion concentration (Supplementary Figure S8), indicating that the nature of the ions plays only a minor role. Consistent with this observation, simulations using our final parameters yield osmotic concentrations that are substantially closer to the experimental curves than those obtained with the original CHARMM36m parameters for all counterion combinations tested (Figure 9, right).

### 4.2 Relaxation Times of Lys-Ser Dipeptides

Next, we compared the simulated dynamics to NMR relaxation data, using both our generated parameters with CHARMM TIP3P and the original CHARMM36m parametrization in combination with the water models CHARMM TIP3P, TIP4P, and OPC. Therefore, we have measured and simulated the capped dipeptide KpS containing a positively charged lysine and a phosphorylated serine in two different charge states: Ac–K^+^-pS^0^-NH_2_ with a neutral phosphate group (SP0) and Ac–K^+^-pS^–1^-NH_2_ carrying a negatively charged phosphate^–1^ (SP1). For comparison, we have also included the unphosphorylated KS dipeptide Ac–K^+^-S-NH_2_. The relaxation times (*T*_1_ and *T*_2_) measured for all NMR-accessible carbons (^13^C) or nitrogens (^15^N), and calculated from MD trajectories, directly depend on the underlying dynamics of the molecules, with the two magnetic fields (600 and 850 MHz, respectively) being sensitive to motions on different time scales. A comparison of NMR relaxation times for all three experimentally investigated dipeptide variants can be found in the Supplementary Figure S9.

Figure 10 summarizes the results for the dipeptide Ac–K^+^-pS^0^-NH_2_ and shows that the original CHARMM36m parameters yield *T*_1_ relaxation times roughly two to three times higher than the experimental values and *T*_2_ values being significantly overestimated. Qualitatively, the simulations show similar patterns like the experimental values, i.e., the relaxation times of nitrogen atoms are higher than those of the carbon atoms. Our parameters (MP0-MP0 vpc) just very slightly improve the results. For the dipeptide Ac–K^+^-pS^–1^-NH_2_, shown in Figure 11, similar general trends are observed. Most parameter sets from force matching do not improve the overestimation of the relaxation times from simulation, only Na^+^ vc and vpc relaxation times are minimally lower than those obtained with the original CHARMM36m parameters. Strikingly, the water model has a much greater impact on the relaxation times than the phosphate parameters, as using TIP4P or even better OPC yields relaxation times much closer to the experimental ones than all the simulations with CHARMM TIP3P. Puzzled by this disagreement, we have studied relaxation times in experiment and simulations also for the unphosphorylated form of the dipeptide, i.e., Ac–K^+^-S-NH_2_, which revealed a similar mismatch between the methods like Ac–K^+^-pS^0^-NH_2_ (see Supplementary Figure S10), showing that the CHARMM36m force field in combination with the CHARMM TIP3P water model poorly reflects the dynamics of the studied solvated dipeptides.^110–113^ Such a mismatch was also reported before for the proteins HpTonB-92 and PaTonB-96 with TIP3P, TIP4P, and OPC in the literature.^77^ Moreover, it has been previously shown that the water model has a significant impact on rotational dynamics in simulation of the intrinsically disordered protein COR15A.^78^ The comparison of the experimental data for Ac–K^+^-pS^–1^-NH_2_ and Ac–K^+^-S-NH_2_ (Supplementary Figure S9) shows that the introduction of the -PO_4_^–1^ group only slightly affects the measured NMR relaxation times, which indicates that phosphorylation has a small influence on the overall dipeptide dynamics at pH 4.5.

**Figure 10:**
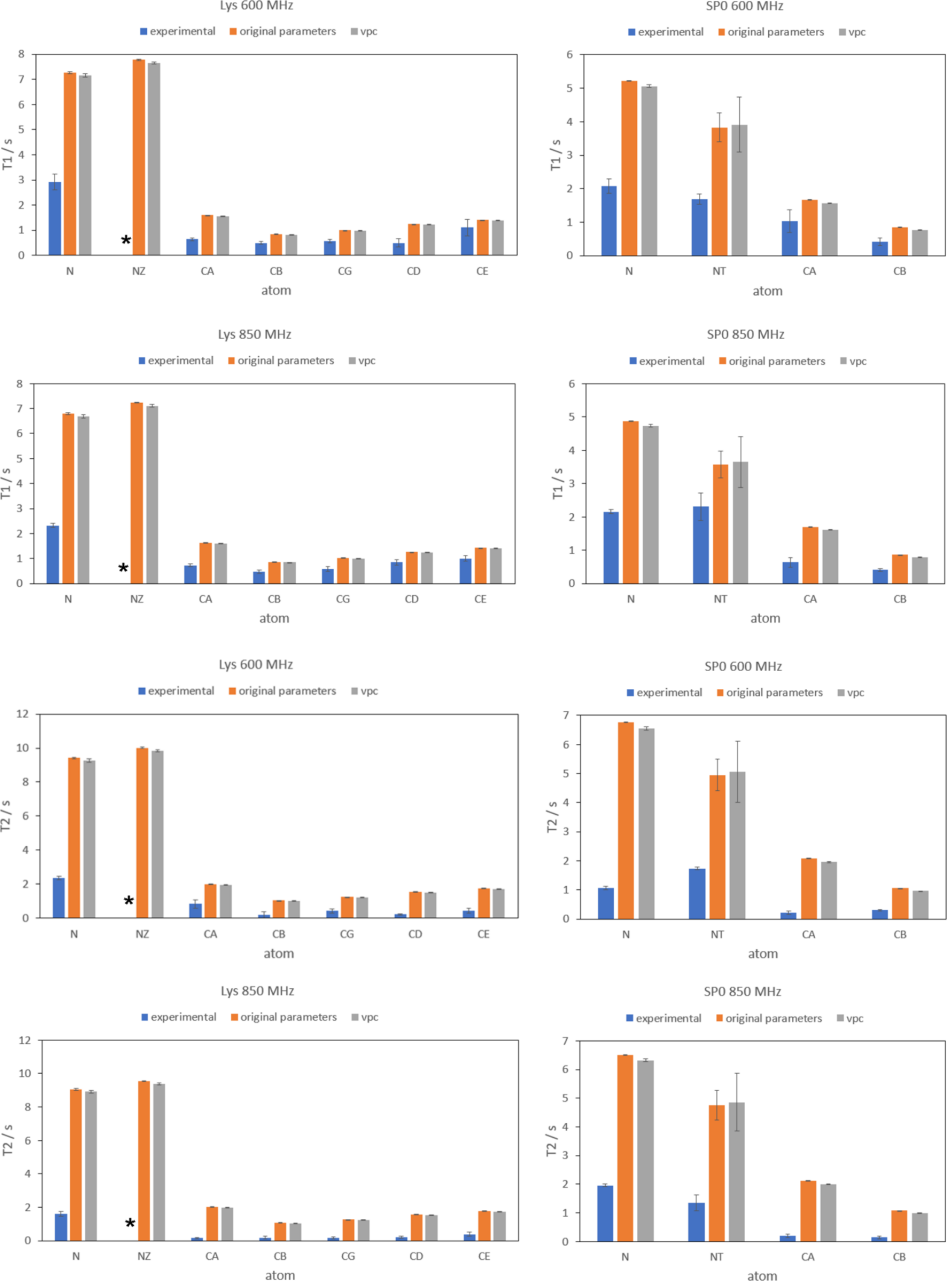
Comparison of NMR relaxation times *T*_1_ and *T*_2_ between experiments and simulations for Ac–K^+^-pS^0^-NH_2_. SP0 is the residue name of phosphoserine^0^. For mapping of the atom names, consult Figure 4. The * in the plot marks relaxation times that could not be measured experimentally. The error bars denote standard error of the mean estimated over 3 independent dipeptides in MD simulations and standard errors from the peak fit of the experimental resonances according to eq 7. The experiment and simulation were conducted at 298 K.

**Figure 11:**
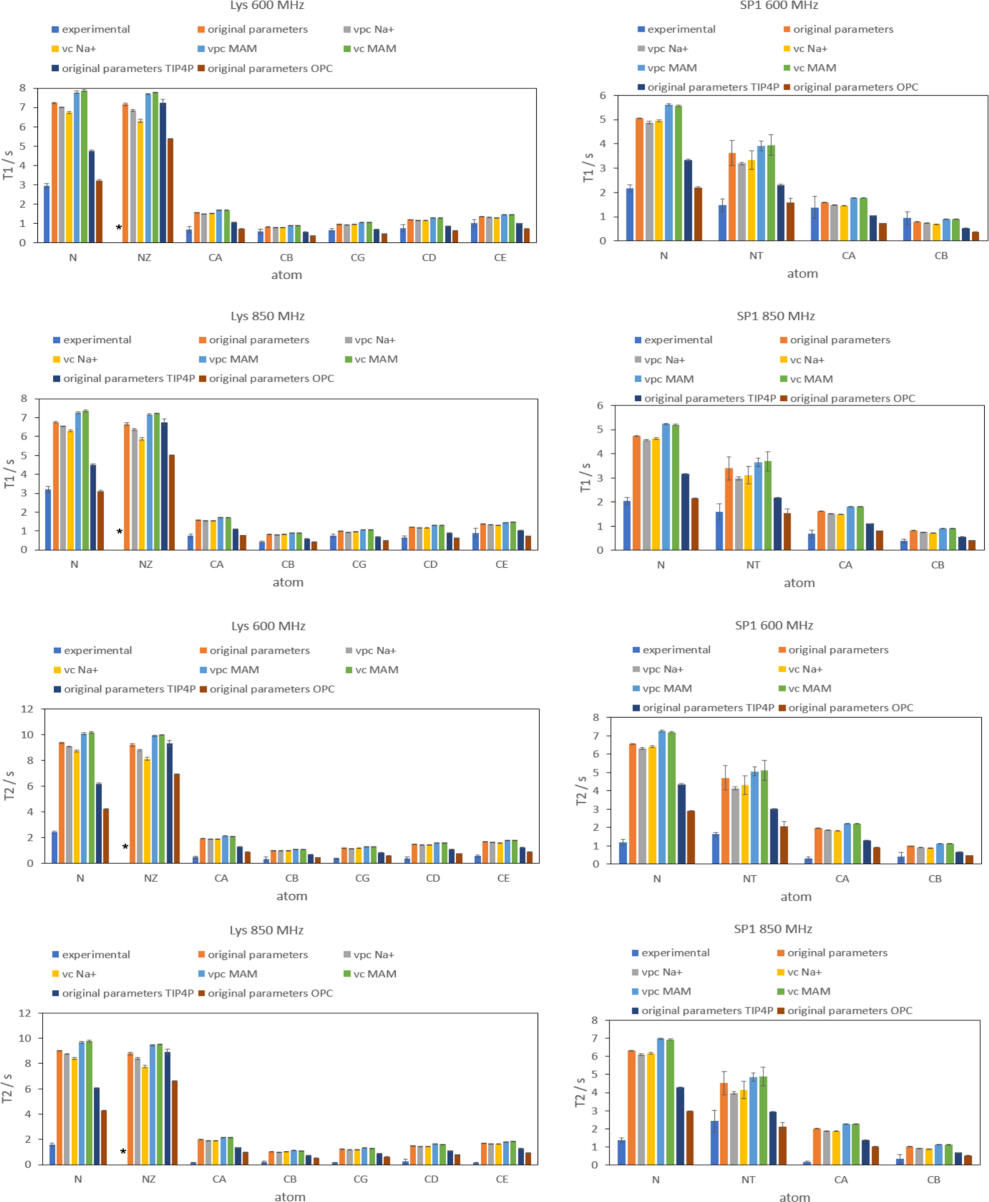
Comparison of NMR relaxation times *T*_1_ and *T*_2_ between experiments and simulations for Ac–K^+^-pS^–^-NH_2_. SP1 denotes the residue name of phosphoserine^–1^. For mapping of the atom names, refer Figure 4. The * in the plot marks relaxation times that could not be measured experimentally. The error bars denote standard error of the mean estimated over 3 independent dipeptides in MD simulations and standard errors from the peak fit of the experimental resonances according to eq 7. The experiment and simulation were conducted at 298 K.

### 4.3 Protein Simulations

To evaluate our parameters in a protein context, we conducted 1 μs-long simulations of the anti-sigma factor antagonist SpoIIAA in its unphosphorylated form (pdbID 1H4Y,^45^ visualized in Supplementary Figure S11) and with a phosphorylated serine (pS57) (pdbID 1H4X,^45^ visualized in Supplementary Figure S12). The phosphorylated serine was simulated in two different protonation states, namely, -PO_4_H^–1^ (‘P1’) or -PO_4_^–2^ (‘P2’) using either the original CHARMM36m parameters or our final parameters generated for MP1 and MP2 (Table 2). All parameters yielded stable protein simulations with root-mean-square deviations (RMSD) of the protein backbone fluctuating between 1.2 and 2.3 Å and no systematic drift over time (see Figure 12).

**Table 2:** Original and Final MP Parameters^a^

		**original parameters**			**final parameters**		
**force-matching system**	**atom type**	**charge**	σ**/nm**	ε ***/*** **kJ**·**mol** ^–1^	**charge**	σ**/nm**	ε**/kJ**·**mol** ^–1^
MP0-	OPM0	–0.560	0.294	0.418	–0.530	0.299	2.045
MP0	PON	1.493	0.383	2.448	1.980	0.432	0.101
vpc	OPH0	–0.622	0.314	0.804	–0.800	0.305	0.776
	OP0	–0.642	0.303	0.502	–0.910	0.291	1.807
	HOP0	0.420	0.040	0.192	0.480	0.030	0.132
MP1-	OPM1	–0.621	0.294	0.418	–0.640	0.325	0.399
MAM	PO1	1.500	0.383	2.448	1.640	0.246	1.277
vc	OPH1	–0.671	0.314	0.804	–0.740	0.543	0.200
	OP1	–0.823	0.303	0.502	–0.790	0.307	0.850
	HOP1	0.338	0.040	0.192	0.220	0.059	0.097
MP2-	OPM2	–0.399	0.294	0.418	–0.680	0.570	0.002
MAM	PO2	1.099	0.383	2.448	1.750	0.056	7.990
vc	OP2	–0.900	0.303	0.502	–0.690	0.285	1.387

a ε and σ parameters are rounded to 6 decimal digits. For full accuracy, see the ffnonbonded.itp file in charmm36-jul2022mod.ff in the accompanying DaRUS repository or in the Supplementary Table S4. MP0 stands for methylphosphate^0^, MP1 for methylphosphate^–1^, MP2 for methylphosphate^–2^, and MAM for methylammonium^+^, while vc stands for variance over all conformations. Mapping of the here introduced atom types can be found in Figure 5.

**Figure 12:**
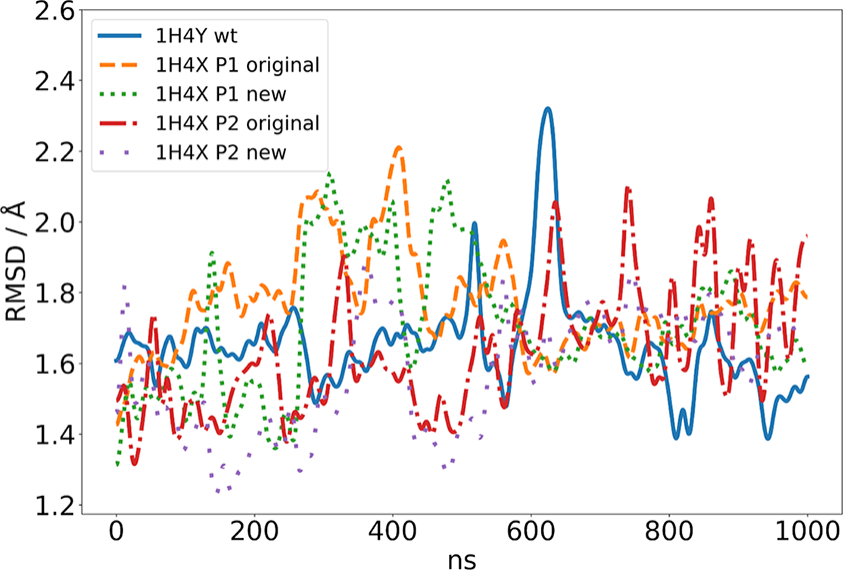
RMSD of C_α_ atoms of the anti-sigma factor antagonist SpoIIAA in its unphosphorylated form (1H4Y wt) and with a phosphorylated serine (pS57)­(1H4X), containing either a singly charged phosphate group (P1) or a doubly charged phosphate group (P2). ‘Original’ denotes the original CHARMM36m parameters, ‘new’ refers to our final parameters for MP1 and MP2 listed in Table 2. The curves have been smoothed using SciPy’s gaussian_filter1d with sigma = 50. As reference structure, the crystal structure of 1H4Y was used.

Secondary structure content analysis using DSSP revealed that the amount of secondary structure elements is comparable across all simulated states of the anti-sigma factor antagonist SpoIIAA. The system that differs most, according to Figure 13, is 1H4X with the doubly charged phosphate (P2) simulated with the original CHARMM36m parameters. It exhibits the smallest overall number of structured residues. Detailed DSSP plots can be found in the Supporting Information Figures S13–S17.

**Figure 13:**
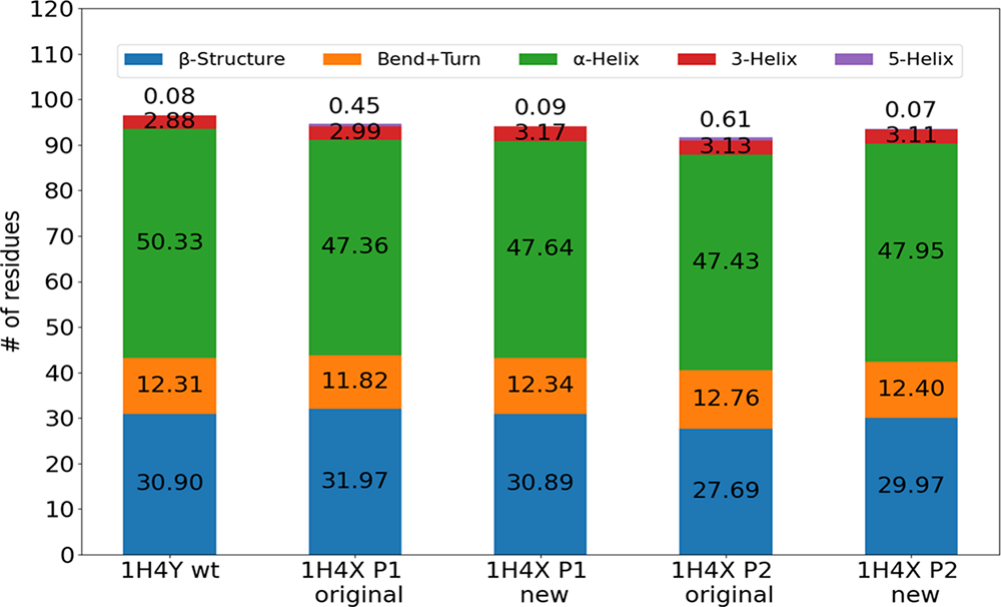
Secondary structure content analysis (DSSP) of 1H4Y (unphosphorylated anti-sigma factor antagonist SpoIIAA) and 1H4X (phosphorylated form with phosphate on serine 57, pS57), containing either a singly charged phosphate group (P1) or a doubly charged phosphate group (P2). ‘Original’ denotes the original CHARMM36m parameters, ‘new’ refers to our final parameters for MP1 and MP2 listed in Table 2. Average over the whole simulation time of 1 μs is given, the topmost values correspond to the 5-Helix content. Coils were omitted for clarity.

Next, we scanned the entire simulation trajectories for residues within a 3.5 Å radius around residue S57/pS57 that could possibly interact with the serine/phosphorylated serine and calculated the root-mean-square fluctuations (RMSF) for all protein simulation systems, revealing that our parameters for MP1 increase the mobility between residues 20 and 40 (peaking at I37). Moreover, both P2 simulations exhibit slightly higher fluctuations of residues immediately preceding pS57 and our final P1 parameters give fluctuations comparable to the wild-type protein, while the original P1 parameters show decreased mobility in this region. Interestingly, the unphosphorylated wild type shows substantially higher fluctuations of residue V8 (see Figure 14). As (p)­S57 and V8 are localized on completely opposite sides of the protein and about 3 nm apart, we assume that the increased flexibility of V8 in the 1H4Y wt simulation results from less tight contacts of V8 with surrounding hydrophobic residues I108 and V14 in the crystal structure rather than from the direct influence of the phosphorylation. Notably, despite the two variants exhibiting a very similar intrachain fold, the two crystals differ substantially in the crystal unit cell size and in the relative orientation of the two copies of the protein inside.

**Figure 14:**
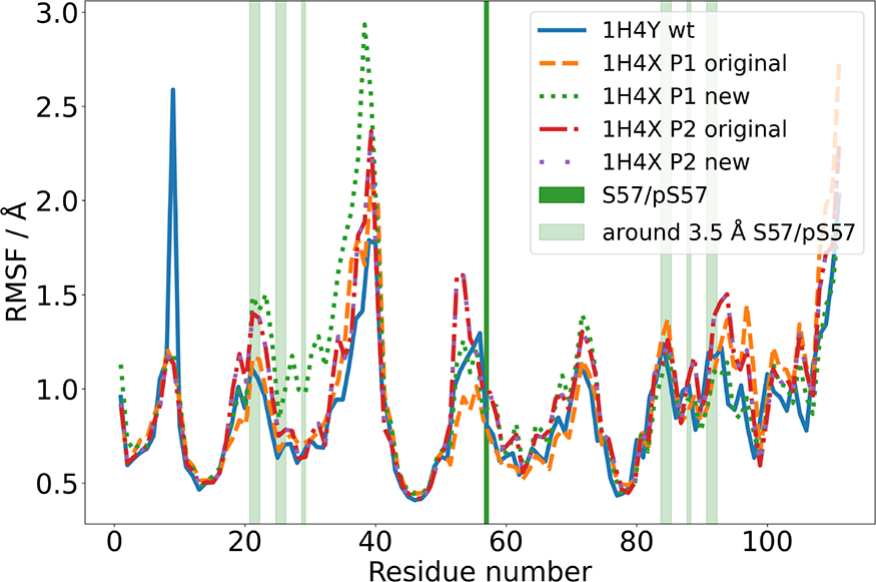
RMSF of C_α_ atoms of the anti-sigma factor antagonist SpoIIAA in its unphosphorylated form (1H4Y wt) and with a phosphorylated serine (pS57)­(1H4X), containing either a singly charged phosphate group (P1) or a doubly charged phosphate group (P2). ‘Original’ denotes the original CHARMM36m parameters, ‘new’ refers to our parameters for MP1 and MP2 listed in Table 2. Residues located in any time point of the simulation within 3.5 Å around the residue 57, i.e., which could interact with S57/pS57, are highlighted in pale green.

Regarding interactions with Na^+^, we observed an average of 0.040 sodium ions within 0.3 nm of all atoms in the pS residue in the simulation with the singly charged pS57 using the original CHARMM36m parameters and 0.036 using our vc MP1-MAM parameters. For the doubly charged pS57, the original parameters resulted in an average of 1.160 Na^+^. Thereby, 27% of the simulation time 2 Na^+^ ions and 3% of the simulation time even 3 Na^+^ ions directly interacted with pS57, reflecting the clumping of MP2 and positively charged ions in the osmotic pressure simulations. In contrast, our final parameters yield an average of 0.027 sodium ions in direct interaction with the fully deprotonated phosphorylated serine residue, resembling the pS/Na^+^ interactions in the singly protonated systems.

## 5 Conclusions

Phosphorylation is a crucial regulatory post-translational modification in biological systems. However, structural and dynamical data of phosphorylated proteins at atomistic resolution are rarely available. Therefore, force field parameters for charged phosphate groups on amino acid residues are often incompletely or inaccurately parametrized and poorly validated. This resulting gap in simulation accuracy not only limits research into biological regulatory processes but also restricts a general understanding of the protein phosphorylation mechanisms. To refine the nonbonded force field parameters of phosphorylated amino acids compatible with the widely used CHARMM36m force field, we have implemented a force-matching procedure against our own quantum chemical calculations of water-solvated amino acid analogs. In the next step, the newly established parameters were thoroughly tested against our own experimental data including osmotic concentrations of methylphosphate/methylammonium solutions and NMR relaxation times of a (phosphorylated) KS dipeptide. Using this approach, we were able to refine nonbonded parameters for fully protonated, singly deprotonated, and doubly deprotonated phosphate groups of methylphosphate, a side chain analog of phosphorylated serine. The new parameters closely match experimentally measured osmotic concentrations, display physical behavior in the hydration shells, and solve the problem of ion/ion overbinding in solution and, consequently, also with phosphorylated proteins. Moreover, molecular dynamics simulations of the anti-sigma factor antagonist SpoIIAA reveal that both the wild-type protein and its phosphorylated variant remain in good agreement with the corresponding X-ray structures, supporting the ability of the refined parameters to accurately describe proteins in their phosphorylated form in different protonation states.

Notably, the total charge of the fully deprotonated phosphate group had to be scaled down from −2 to −1 to achieve solute–solute interactions that reproduce correct osmotic concentrations. Such an adjustment is increasingly applied in classical force fields to account for the missing electronic polarization and to effectively reflect the reduced charge of the solute in its hydrated state.^114–118^ It has been shown in the literature that MP2 is better hydrated than MP1.^119^ Interestingly, our force-matching procedure independently predicted a charge of −0.85 for the fully deprotonated phosphate group, in close agreement with the −1 value used in our final parameters.

Systems combining our methylphosphate parameters with the original CHARMM36m ion parametrization reproduced experimental osmotic concentrations and maintained structural stability in protein simulations, demonstrating the compatibility of both parameter sets. In contrast, evaluation against NMR relaxation times revealed remaining deficiencies in the CHARMM36m amino-acid parameters. In detail, even for the unphosphorylated KS dipeptide, the original CHARMM36m parameters do not correctly capture experimental rotational dynamics, as illustrated in Supplementary Figure S10 and in the literature.^77^ For the backbone and the side chains of both dipeptide residues, the values from simulation are higher than the experimental values. The addition of the charged phosphate group to the side chain of a serine residue only marginally alters the rotational dynamics of the backbone. Therefore, modifying the parameters of the phosphate group in KpS cannot drastically improve the agreement of the simulated and experimental relaxation times. On the contrary, substantial improvement of the rotational dynamics in phosphorylated peptides after phosphate reparametrization would hint to error cancellation rather than resulting from a physically improved description. Exchanging the water model to TIP4P or OPC has a greater impact improving the agreement with experiments, especially for OPC. However, it has to be noted that the compatibility of the CHARMM36m force field with the OPC water model has not been thoroughly tested, even though first comparisons do exist.^120^ In agreement with the role of the water model for peptide rotational dynamics, our osmotic concentration calculations also demonstrate that the choice of the water model influences the output of MD simulations, yet it cannot compensate for inaccurate solute–solute parameters.

Notably, our force-matching Python package The Phorce offers a flexible parametrization tool with an interface to cp2k, a state-of-the-art QC package, as well as to OpenMM, which supports various force fields. On top of that, we share our QC calculations of forces in between diverse amino acid analogs and ions together with The Phorce, which opens up avenues to use our force-matching strategy for the parametrization of other force fields or for different solutes.

## Supplementary Material



## Data Availability

The force field files (for GROMACS and CHARMM simulation engines), MD simulation run files, cp2k input files, and snapshots and trajectories of the simulations will be made available online at DaRUS at 10.18419/darus-5682, and the results of the QC calculations will be made freely accessible at Zenodo at 10.5281/zenodo.17966358 upon publication. The experimentally measured values are provided in tabular form in Excel sheets included in nmr_MD_sims.zip (in detail in the file relaxation_times.xlsx) and osmotic_experimental.zip at DaRUS at 10.18419/darus-5682. The force-matching package The Phorce is available on GitHub at https://github.com/CornyC/The_Phorce.git. Jupyter notebooks for analysis are available at DaRUS at 10.18419/darus-5682, https://github.com/CornyC/Box_Slicer, and https://github.com/CornyC/Osmotic_pressure_simulations_w_Gromacs_pull_code.

## Notes

A description of The Phorce package, a table of the simulated methylphosphate systems, a table with the charge variations tested for MP2, a visualization of the original CHARMM36m parameters MP2–2MAM osmotic pressure simulation box, and a table with full-precision values of our parameters; next, a section discussing the manual adjustments of nonbonded parameters, a comparison of experimental *c*_osm_ of MPA with MAM to experimental and literature values of NaCl, a table of the pH values of the measured MPA-MAM solutions, the influence of different NBFIX on *c*_osm_, and a comparison of *c*_osm_ of the new Amber parameters for phosphorylation to experimental values, original CHARMM36m parameters and our parameters, followed by RDFs for MP2^–1^-1MAM and a table of the chemical shifts of KpS, a comparison of experimental relaxation times of KS and KpS at pH = 2 and pH = 4.5, and relaxation times for KS; and images of 1H4Y and 1H4X together with the detailed results of the DSSP analyses (PDF)
