# Supplementary material for: Evaluating Force Matching as a Parametrization Strategy for the CHARMM36m Force Field Using Phosphorylation
Source: J Phys Chem B. 2026 May 22;130(22):5469–87. doi: 10.1021/acs.jpcb.6c00779 (PMC13244478; doi:10.1021/acs.jpcb.6c00779)
Supplement: Supplementary file 1 [file jp6c00779_si_001.pdf]

Supporting Information: Evaluating Force Matching as a Parametrization Strategy for the CHARMM36m Force Field using Phosphorylation

Viktoria Korn,[†] Tobias Rindfleisch,[‡,¶,§] Sandra Posch,[‖] Jarl Underhaug,[¶] Andreas Horner,[‖] Markus Miettinen,[‡,¶] and Kristyna Pluhackova[*,†]

†*Stuttgart Center for Simulation Science, Cluster of Excellence EXC 2075, University of Stuttgart, 70569 Stuttgart, Germany*

‡*Computational Biology Unit, Department of Informatics, University of Bergen, 5008 Bergen, Norway*

¶*Department of Chemistry, University of Bergen, 5007 Bergen, Norway*

§*Max Planck Institute of Molecular Plant Physiology, 14476 Potsdam, Germany*

‖*Institute of Biophysics, Johannes Kepler University Linz, 4040 Linz, Austria*

E-mail: kristyna.pluhackova@simtech.uni-stuttgart.de

The Phorce - A Python Package for Force-Matching

`The Phorce` is a Python package developed for processing all input and output data to and from the objective function. `The Phorce` is available on GitHub at https://github.com/CornyC/The_Phorce.git. The user interface is based on the jupyter notebook The_Phorce _UI.ipynb, providing flexibility while also guiding the process of parametrization. The `Paths` module registers input and output directories and file names and a MDAnalysis[1,2] interface can be used to prepare coordinate files for the net force calculations. It also handles pdb and trajectory file outputs as well as coordinate extractions. If desired, DFT calculations can be directly initiated from the package via an ASE[3] interface, offering numerous methods, or via a cp2k interface (which requires a local installation of cp2k). Both single-point calculations and geometry optimizations are possible together with automatic extractions of forces, energies, charges, and optimized coordinates in case of geometry optimizations. For computationally expensive systems, it is also possible to read in the respective outputs from ASE or cp2k if the calculations were run on an HPC cluster. Central data management and storage is handled by the `System` module, including the calculation of net forces that remove e.g. solvent interactions from the parametrization process. Classical calculations are done via an OpenMM[4] interface which can handle all sorts of common MD file types and force fields, including those from CHARMM, GROMACS, and NAMD. The set of optimizable parameters can be constructed from a topology-based atom selection and may include both bonded or non-bonded parameters. To streamline the optimization, an automatic parameter reduction is executed which removes parameters from duplicate atom types from the set of optimizable parameters. For the CHARMM-type force fields, this reduction also works with NBFIXes. The unique parameter set is then vectorized and scaled using Z-scores to guarantee an equal handling of all parameters, despite their different types and magnitudes. If desired, weights per conformation can be added by the user. The `Optimizer` module provides a wrapper for local and global SciPy optimizers,[5] pyCMA[6] (a stochastic optimizer for robust non-linear non-convex derivative-free and function-value-free numerical optimization), BOSS[7] (a Bayesian optimizer), and a homemade particle swarm optimizer. It also handles the penalty function and all optimizer settings are accessible through it. If certain bounds (set automatically or manually, depending on the specific property) are violated, a number-of-optimizer-iterations-based penalty is added to the objective function value. The `Parametrization` module evaluates the objective function including weights and bounds, wraps it in an optimizer-compatible way, and runs the optimization. If the optimization was successful, a set of optimized parameters together with the respective atom types is returned. After testing several optimizers, SciPy's local Nelder-Mead[8] with a tolerance of $1 \cdot 10^{-5}$, bounds, and automatically adapted algorithm parameters combined with an additional penalty term lead to the fastest and most applicable results for our cases. The constraints used for the optimizer's native bounds as well as the penalty term kept

the nonbonded parameters (charges and Lennard-Jones (LJ)) within ranges summarized in Table S2.

Table S1: Simulated methylphosphate systems for extraction of conformations for DFT calculations of intermolecular forces.

**Methylphosphate**	**Charge of the phosphate group**	**(Cat)ion**	**Mixing ratio**
2 methylphosphate (MP0)	±0		
methylphosphate (MP0)	±0	Cl^- + methyl-ammonium (MAM)	1:1:1
methylphosphate (MP1)	−1	Na^+	1:1
methylphosphate (MP1)	−1	methylammonium (MAM)	1:1
methylphosphate (MP2)	−2	Na^+	1:2
methylphosphate (MP2)	−2	methylammonium (MAM)	1:2

Table S2: Boundaries used in the optimization of the here studied systems. Elementary charge is given and the units of σ and ε are given only for maxima values.

**Charge**			**LJ parameters**		
**atom**	**min**	**max**	**type**	**min**	**max**
H	0.0	1.0	σ	0.03	1.0 nm
C	-4.0	4.0	ε	0.002	20 kJ/mol
O	-2.0	0.0	NBFIX r_{min}	0.2	1.0 nm
P	0.0	6.0	NBFIX ε	0.06	15.5 kJ/mol

Table S3: Atomic charge variations for parameter sets of MP2. Corr. indicates charge correction of the parameters to the total charge -2 of the phosphate group. Total denotes the total charge of the phosphate group.

	**Charge**						
**Atom type**	**MP2-2MAM vpc**	**MP2-2MAM vpc charge corr.**	**MP2-2MAM vc**	**MP2-2MAM vc charge corr.**	**vc original charge P**	**vc original charge O**	**vc total charge -1**
OPM2	-0.616	-0.850	-0.653	-0.880	-0.930	-0.650	-0.680
PO2	1.720	1.490	1.779	1.550	1.780	0.630	1.750
OP2	-0.646	-0.880	-0.662	-0.890	-0.950	-0.660	-0.690
Total	-0.834	-2	-0.86	-2	-2	-2	-1

Table S4: Input and output force field parameters from The Phorce. MP0 stands for methylphosphate^0, MP1 for methylphosphate^{-1}, MP2 for methylphosphate^{-2}, MAM for methylammonium$^+$. SOD is CHARMM36 atom type of Na^+. # indicates NBFIX parameters, vpc stands for variance per conformation and vc for variance over all conformations. Iters gives the number of iterations performed by the force fitting algorithm. Final charge denotes charges after charge correction (see Main Text Section 2).

		**Input (original) parameters**			**Output parameters**				
**Simulation System**	**Atom type**	**Charge**	σ **/ nm**	ε **/ kJ·mol^{-1}**	**Charge**	**Final charge**	σ **/ nm**	ε **/ kJ·mol^{-1}**	**Iters**
MP0-	OPM0	-0.560	0.293996576986	0.4184000	-0.541	-0.530	0.298616061916	2.0449074	10606
MP0	PON	1.493	0.383086448800	2.4476400	1.969	1.980	0.432010436130	0.1013734	
vpc	OPH0	-0.622	0.314487247504	0.8037464	-0.806	-0.800	0.304945764711	0.7759720	
	OP0	-0.642	0.302905564168	0.5020800	-0.916	-0.910	0.290582617359	1.8070626	
	HOP0	0.420	0.040001352445	0.1924640	-0.471	0.480	0.030002586326	0.1316080	
MP1-	OPM1	-0.621	0.293996576986	0.4184000	-1.014	-1.210	0.361365612064	0.5658485	3025
Na	PO1	1.500	0.383086448800	2.4476400	3.459	3.260	0.477238085992	2.8572228	
vc	OPH1	-0.671	0.314487247504	0.8037464	-0.684	-0.880	0.510841217150	0.0020522	
	OP1	-0.823	0.302905564168	0.5020800	-0.944	-1.140	0.296709819194	1.6122998	
	HOP1	0.338	0.040001352445	0.1924640	0.000	0.010	0.032292774719	0.1194490	
	SOD/OP1#		0.316000000000	0.3138837			0.645166003681	0.4586287	
MP1-	OPM1	-0.621	0.293996576986	0.4184000	-0.938	-1.100	0.332875044854	1.0735146	6835
Na	PO1	1.500	0.383086448800	2.4476400	2.910	2.750	0.685450055358	0.0071338	
vpc	OPH1	-0.671	0.314487247504	0.8037464	-0.397	-0.560	0.505416022356	0.0020004	
	OP1	-0.823	0.302905564168	0.5020800	-0.953	-1.100	0.290161229224	1.4620632	
	HOP1	0.338	0.040001352445	0.1924640	0.008	0.010	0.077229979532	0.0488784	
	SOD/OP1#		0.316000000000	0.3138837			0.646461996325	1.3292413	
MP1-	OPM1	-0.621	0.293996576986	0.4184000	-0.582	-0.640	0.324639930419	0.3988666	3334
MAM	PO1	1.500	0.383086448800	2.4476400	1.692	1.640	0.245781549707	1.2771433	
vc	OPH1	-0.671	0.314487247504	0.8037464	-0.694	-0.740	0.543133180526	0.2000239	
	OP1	-0.823	0.302905564168	0.5020800	-0.743	-0.790	0.306965696556	0.8501271	
	HOP1	0.338	0.040001352445	0.1924640	0.268	0.220	0.059041310910	0.0965576	
MP1-	OPM1	-0.621	0.293996576986	0.4184000	-0.560	-0.630	0.337665605364	0.2446355	2745
MAM	PO1	1.500	0.383086448800	2.4476400	1.596	1.530	0.268253709335	5.2822660	
vpc	OPH1	-0.671	0.314487247504	0.8037464	-0.572	-0.640	0.469191978208	0.0071889	
	OP1	-0.823	0.302905564168	0.5020800	-0.712	-0.770	0.312376580633	0.6440177	
	HOP1	0.338	0.040001352445	0.1924640	0.255	0.180	0.084281421764	0.1858065	
MP2-	OPM2	-0.399	0.293996576986	0.4184000	-0.653	-0.880	0.569557817706	0.0020092	1649
2MAM	PO2	1.099	0.383086448800	2.4476400	1.779	1.550	0.055727625216	7.9902009	
vc	OP2	-0.900	0.302905564168	0.5020800	-0.662	-0.890	0.284801626767	1.3867718	
MP2-	OPM2	-0.399	0.293996576986	0.4184000	-0.616	-0.850	0.401940430930	1.0989495	1421
2MAM	PO2	1.099	0.383086448800	2.4476400	1.720	1.490	0.267134632621	11.4715621	
vpc	OP2	-0.900	0.302905564168	0.5020800	-0.646	-0.880	0.278455018845	1.9988805	

Manual Variation of the Nonbonded Parameters of the Phosphate Group in MP2

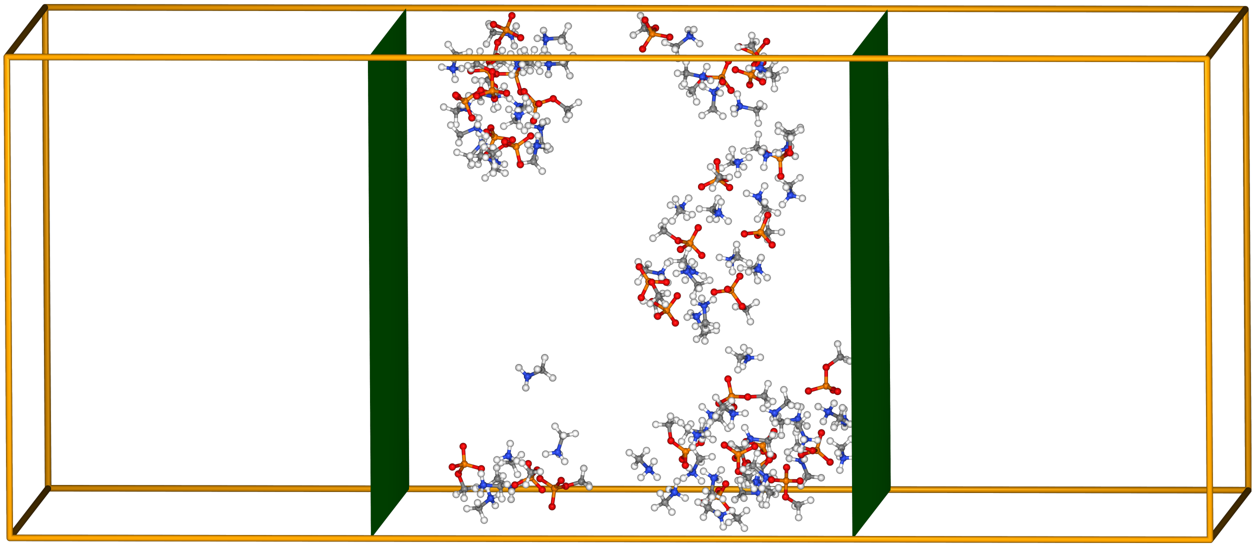

Figure S1: Simulation box for osmotic pressure estimation of MP2-MAM 1:2 solution in a total concentration of 1.2 mol/L using the original CHARMM36m parameters and the CHARMM TIP3P water model. Water has been omitted from visualization for clarity. The two force boundaries which are acting only on solutes, i.e., on MP2 and MAM, are visualized as green surfaces. The z-axis is located in the left-right direction. The solutes are shown in ball+stick representation, hydrogen atoms are colored white, oxygens red, phosphorus atoms orange, and nitrogens blue.

At first we varied the Coulomb parameters in our MP2–2MAM vc parameter set. It is important to recall, that for compatibility with the CHARMM36m protein parameters (see Figure S2, right), a charge correction was applied to the charges obtained from force matching (see Equation 3 in the main manuscript) balancing the total charge difference equally over all atoms of the phosphate group. To investigate the influence of the atomic charges on the osmotic concentration, the total charge of the phosphate group was tentatively corrected by taking the charges of the phosphate oxygens as predicted by `The Phorce` and adapting the charge of the phosphorus only (see Figure S2, parameter set "oc oxygens" shown as red circles, for the resulting osmotic concentrations). Alternatively, the total charge of the phosphate group was adjusted by altering the oxygen charges and keeping the phosphorus charge as predicted by `The Phorce` (shown in Figure S2, as the parameter set "oc phosphorus" as orange squares). An overview of the tested charges is listed in Supplementary Table S3. Figure S2 shows that distributing the charges differently has only a small effect on the osmotic concentration. The same applies to changing the Lennard-Jones parameters of the phosphate group to those obtained by force matching for MP1, i.e., to the LJ parameters utilized

in the MP1–MAM vc parameter set (Figure S2, "mp1mam_vc_LJ" parameter set shown as teal stars) or combining both (Figure S2, "mp1mam_vc_LJ + oc oxygens" parameter set visualized as dark blue triangles).

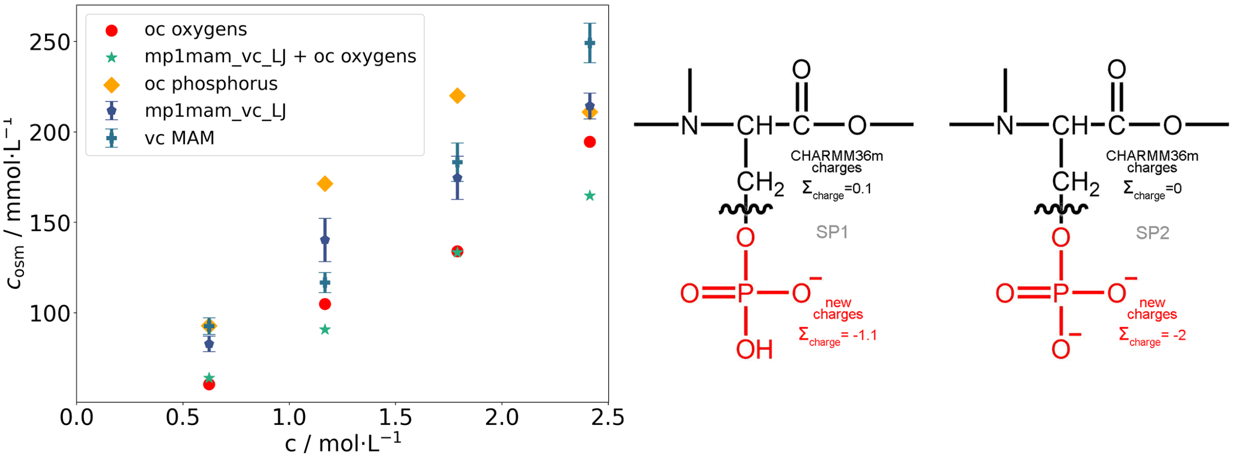

Figure S2: Left: Influence of manual modifications of the MP2–2MAM vc parameter set (vc MAM, blue line) on the osmotic concentration: Correcting the group charge by adapting the charge of the phosphorus only (oc oxygens, red circles), or by adjusting the total charge through the oxygens (oc phosphorus, orange squares), using Lennard-Jones parameters from MP1–MAM vc (mp1mam_vc_LJ, teal stars), and a combination of both (mp1mam_vc_LJ + oc oxygens, dark blue triangles). For test purposes only one simulation per dataset was performed in most cases. For comparison, the experimental osmotic concentration of MPA–MAM 1:2 solution is located outside the y-range and amounts to 514 mmol/L, 1099 mmol/L, and 1324 mmol/L for total concentration of solutes of 0.63 mol/L, 1.26 mol/L, and 1.47 mol/L, respectively. Right: Charges of the backbone including C_β and the phosphate group in the SP1 and SP2 residues for compatibility with the CHARMM36m force field. Our model compounds are MP1 for SP1 and MP2 for SP2. In the black parts, CHARMM36m charges were kept constant while in the red ones, the charges were adapted so that the sum of total charges reached -1 for SP1 and -2 for SP2 residue, respectively.

Because solvation also greatly influences the behavior of ions in solution, as the simulations using distinct water models have shown, we decided to examine the influence of NBFIX applied between the OP2 oxygen atom types of the phosphate group (see Figure 5 in the main manuscript), termed as terminal phosphate oxygens from now on, and both the hydrogen and oxygen atoms of water on the osmotic concentration. Therefore, NBFIX corrections with σ and ε individually increased or decreased by 10–20 % were introduced together with the MP2–2MAM vc parameter set to analyze their effect on the osmotic concentration (Figure S3). In general, no significant change in the osmotic concentration could be achieved by varying the LJ parameters between the terminal phosphate oxygens and water hydrogens. Decreasing σ by 20 % between the terminal phosphate oxygens and the water's oxygens (OT) causes a significant increase of the osmotic concentration as water molecules can spatially

approach the phosphate group more closely (Figure S3, right). Yet, the absolute values are still far away from the experimental osmotic concentrations. Diminishing σ further to -40 % does not improve the agreement between simulated and experimental osmotic concentration.

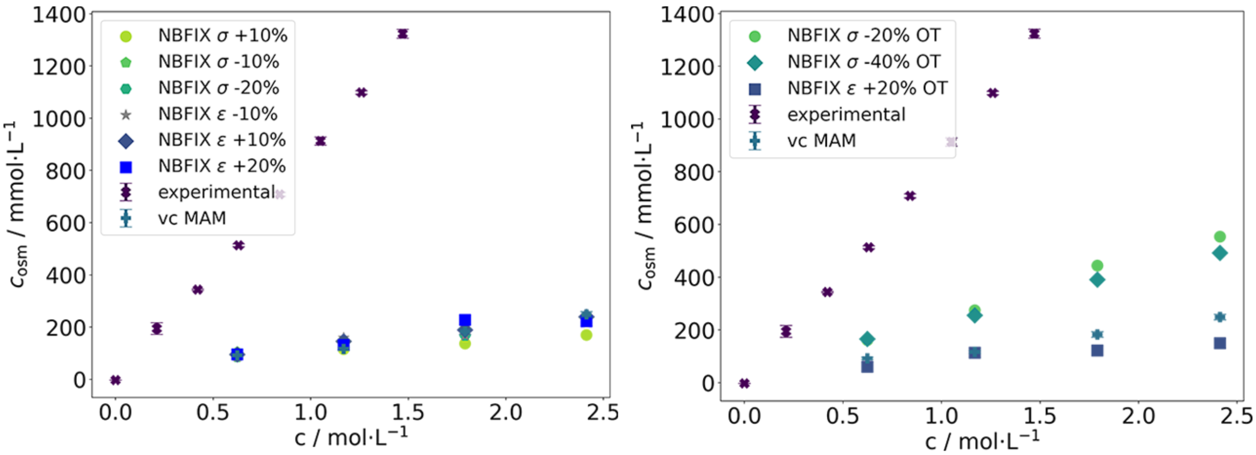

Figure S3: Osmotic concentrations of MP2-2MAM in solution in MD simulations and experiment using MPA-MAM 1:2 solution. Osmotic concentrations are plotted against total molar concentrations of both electrolytes. Left, the effect of NBFIXes applied between the terminal oxygens of the phosphate group and the water's hydrogens. Right, the effect of NBFIXes applied between the terminal oxygens of the phosphate group and the water's oxygens (OT). The generated parameters from MP2-2MAM vc were used as base values. For test purposes only one simulation per dataset at a given concentration was performed. More detailed plots without the experimental data set are in Supplementary Figure S5.

Modifying intermolecular interactions to water interferes with the formation of hydration shells as the water molecules can approach the solute either more closely or not and bind to it with a different strength. The group of Marcus Elstner has calculated H-bonding energies and distances between phosphorylated serine amino acids analogs MP1 and MP2 and water, respectively, using high level QM in vacuo, showing that MP2 interacts much stronger with water than MP1 and the hydrogen bonds are by about 0.2 Å shorter.[9] Ab-initio MD has revealed a slight shift of the position of the first maximum of the P–O(water) RDF for phosphoric acid following its deprotonation from H_3PO_4 over $H_2PO_4^-$ and HPO_4^{2-} to PO_4^{3-} (3.68, 3.71, 3.76, and 3.78 Å, respectively), presumably due to increasing steric hindrance with increasing water content of the first hydration shell. The increasing charge also causes an increase in the number of water molecules in the first solvation shell, and, in agreement with the Hofmeister series, exerts larger structuring effects.[10] Radial distribution functions (RDFs) can be easily calculated from MD simulations to reveal the position and occupancy of hydration shells. For comparison, we have calculated the RDFs of water hydrogen or oxygen atoms around terminal phosphate oxygens of MP2 simulated using (i) original CHARMM36m parameters with the CHARMM TIP3P water model, (ii) the original

CHARMM36m parameters with the OPC water model, (iii) our MP2-2MAM vc parameter set, and (iv) our MP2–2MAM vc parameter set with a NBFIX with a by 20 % decreased σ between the phosphate group's terminal oxygens and the water oxygens (Figure S4).

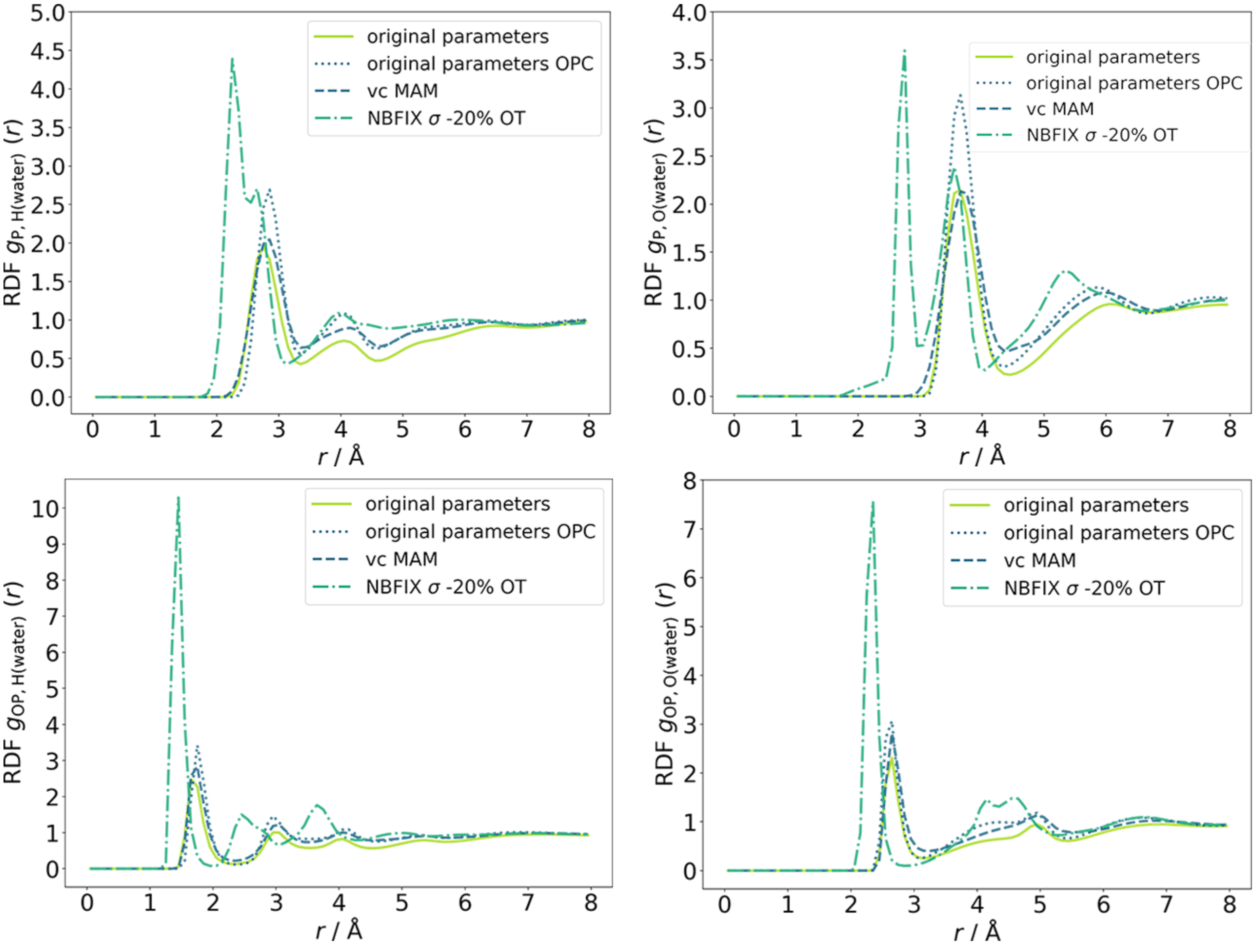

Figure S4: RDFs of water hydrogen or oxygen atoms around the atoms of the phosphate group of MP2, i.e., phosphorus or terminal phosphate oxygens (OP) for different parameter sets and water models. NBFIX σ -20 % OT refers to the MP2–2MAM vc parameter set with an additional NBFIX where σ between the phosphate group's terminal oxygens and the waters' oxygens is decreased by 20 %. The analysis was performed in the system with 1.2 mol/L total solute concentration.

According to quantum-mechanical calculations from the literature,[11,12] the P–H (water) RDF should have its first maximum at approximately 2.8 Å and the second maximum should be located between 4 and 5 Å. This is true for all systems except the one with the NBFIX σ -20 % OT (see Figure S4, top left). For the OP–H (water) RDF, the first peak is expected between 1.5 and 2 Å, the second at approximately 3 Å, and a third one at around 4 Å.[11,12] Again, all systems adequately represent the position of these maxima, except for the NBFIX σ -20 % OT one. The other RDFs in Figure S4 follow the same pattern, showing significantly

distorted hydration shells in the NBFIXed dataset. This misalignment of the peak positions in the NBFIXed dataset exhibits that reducing the effective radius of the phosphate-water interactions is not a physically correct approach to improve the osmotic concentration of MP2–MAM solutions. Additionally, the comparison of the RDFs shows, that the smallest occupancy of the near hydration shells is achieved by using the original CHARMM36m dataset with CHARMM TIP3P water model (light green, solid line), hinting at a generally weaker interaction of water with the phosphate group which causes binding of the phosphate group to other solutes in the system, i.e., MAM or other MP2 molecules.

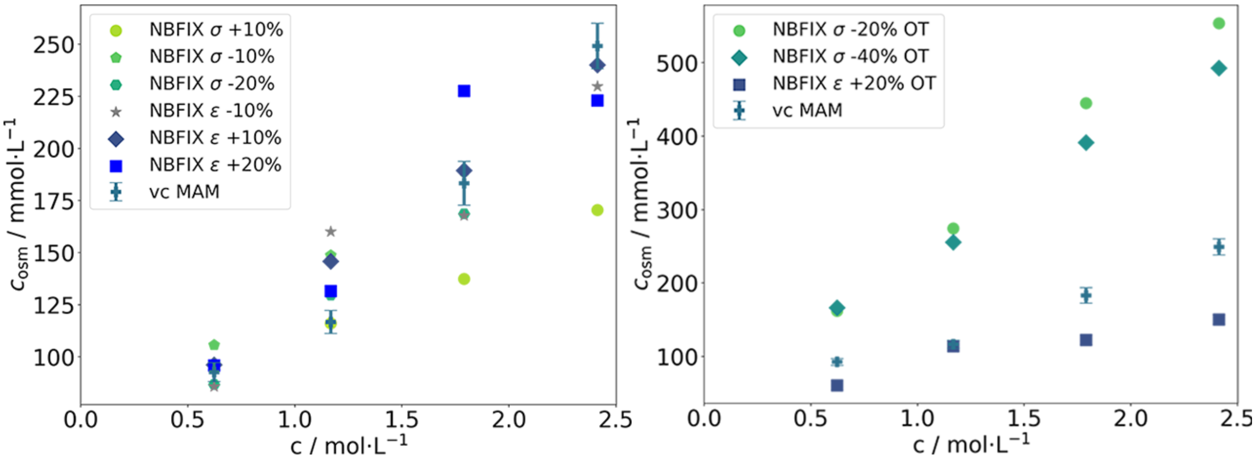

Figure S5: Role of intermolecular Lennard-Jones parameters between the phosphate group and water for osmotic concentrations of MP2-2MAM in solution in MD simulations. Osmotic concentrations are plotted against total molar concentrations of both electrolytes. Left, the effect of NBFIXes applied between the terminal oxygens of the phosphate group and the water's hydrogens. Right, the effect of NBFIXes applied between the terminal oxygens of the phosphate group and the water's oxygens. The generated parameters from MP2-2MAM vc were used as base values. For test purposes only one simulation per dataset at a given concentration was performed.

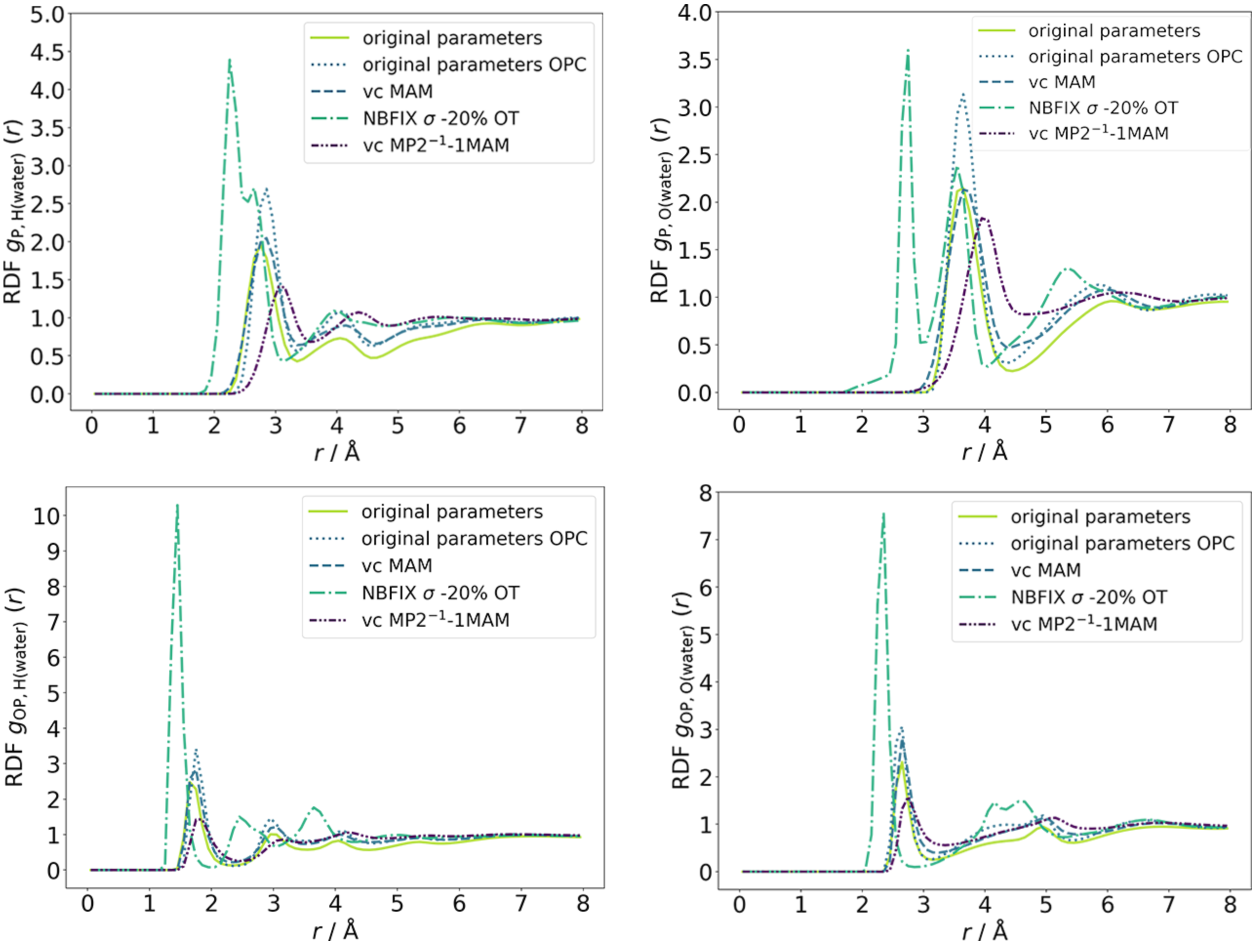

Figure S6: RDFs of water hydrogen or oxygen atoms around the atoms of the phosphate group of MP2, i.e., phosphorus or terminal phosphate oxygens (OP) for different parameter sets and water models. NBFIX σ -20 % refers to the MP2-2MAM vc parameter set with an additional NBFIX where σ between the phosphate group's terminal oxygens and the waters' oxygens is decreased by 20 %. vc MP2^{-1}-1MAM, i.e., our final parameters, has the Lennard-Jones parameters from vc MP2-2MAM but the charge of MP2 is scaled down to -1. The analysis was performed in the system with 1.2 mol/L total solute concentration.

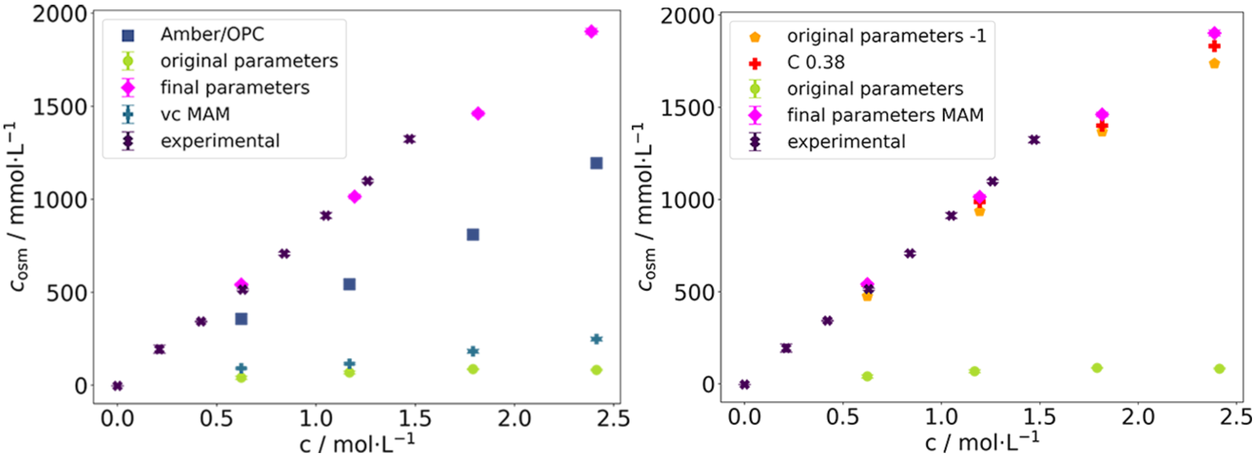

Figure S7: Left: Comparison of the osmotic concentration yielded when using the phosphorylation parameters from the Amber ff19SB force field[13] and OPC water model to experimental values, the original CHARMM36m parameters, the vc MP2-2MAM parameter set, and the $MP2^{-1}$-1MAM parameter set, being our final parameters, (whose Lennard-Jones parameters originate from vc MP2-2MAM). For test purposes only one Amber simulation per data point was performed. Right: The influence of scaling the charge of the phosphate group in combination with the original CHARMM36m parameters for MP2 to -1 (orange dots). For test purposes, only one simulation per data point was performed. In an additional test based on our final parameters for MP2, the charge of the phosphorus atom in the vc $MP2^{-1}$-1MAM was shifted partially to the neighboring carbon, giving it an arbitrary charge of +0.38 (red plus). For test purposes only one simulation per data point was performed.

Additional Experimental Data on Osmotic Pressure Measurements

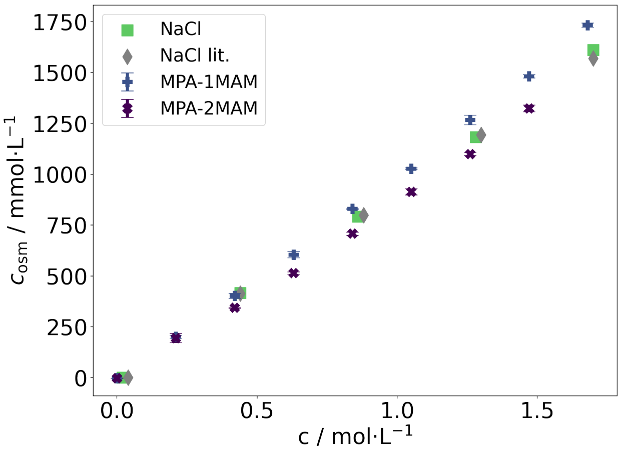

Figure S8: Experimentally measured osmotic concentrations of (i) methylphosphonic acid (MPA) and methylammonium (MAM) 1:2 solution, (ii) MPA-MAM 1:1 solution, (iii) NaCl solution and (iv) literature values for NaCl (CRC Handbook of Chemistry and Physics). For comparability with the other measurements, the total concentration of Na^+ and Cl^- is used, i.e., for example the measured osmotic concentration of 1 M NaCl is plotted against 2 mol/L total concentration, as it consists of two osmotically active particles (Na^+ and Cl^-). The error bars show standard error of the mean over 3 independent replicas. The actual values are provided in the file osmotic_experimental.zip at DaRUS at https://doi.org/10.18419/darus-5682.

Table S5: pH values of experimentally measured MPA-MAM solutions.

1:1 MPA-MAM

**c** / mol/L	**pH**
1	3.36
0.5	3.26
0.25	3.23
0.125	3.27

1:2 MPA-MAM

**c** / mol/L	**pH**
1	7.82
0.5	7.85
0.25	7.92
0.125	7.99

Using the literature pK_a values of the substances at 298 K (MAM 10.66,[14] MPA $pK_a1 = 2.12 - 2.41$ and $pK_a2 = 7.29 - 7.35$[15]) and the Henderson–Hasselbalch relation, the pH values in Table S5 indicate that MAM is almost exclusively in the charged state in both solutions and most of MPA (≈85 %) is in the completely deprotonated state in the MPA-MAM 1:2 solution, while in the MPA-MAM 1:1 solution the majority of MPA is singly protonated (94 %) and the remaining fully protonated.

Supplementary NMR Data and Comparison with MD Estimates

Table S6: NMR chemical shifts of the phosphorylated lysine-serine (KpS) dipeptide under different pH conditions. The N- and C-terminal caps are referenced as ACE and NHE, respectively.

**Residue ID**	**Residue Name**	**Atom Name**	**Chemical Shift / ppm pH 2.0**	**pH 4.5**
1	ACE	C	177.5091	177.5014
1	ACE	CH3	24.5159	24.5249
1	ACE	HH3'	2.0562	2.0563
1	ACE	HH3"	2.0562	2.0563
1	ACE	HH3"'	2.0562	2.0563
2	LYS	N	126.9249	126.9203
2	LYS	CA	56.7344	56.7274
2	LYS	HA	4.3021	4.3076
2	LYS	C	177.2044	177.2077
2	LYS	CB	33.0936	33.0991
2	LYS	HB'	1.8533	1.8556
2	LYS	HB"	1.7843	1.7855
2	LYS	CG	24.5938	24.5866
2	LYS	HG'	1.4683	1.4631
2	LYS	HG"	1.4683	1.4631
2	LYS	CD	29.1464	29.1347
2	LYS	HD'	1.6867	1.6940
2	LYS	HD"	1.6867	1.6940
2	LYS	CE	42.1999	42.1948
2	LYS	HE'	3.0093	3.0089
2	LYS	HE"	3.0093	3.0089
2	LYS	NZ	NaN	NaN
2	LYS	HZ'	NaN	NaN
2	LYS	HZ"	NaN	NaN
2	LYS	HZ"'	NaN	NaN
2	LYS	H	8.3385	8.3349
3	SER	N	116.9775	117.1789
3	SER	CA	56.9714	57.0461
3	SER	HA	4.5415	4.5259
3	SER	C	176.4152	176.4919
3	SER	CB	66.865	66.7496
3	SER	HB'	4.1559	4.1460
3	SER	HB"	4.1559	4.1460
3	SER	P	0.2155	0.4625
3	SER	H	8.5785	8.6094
4	NHE	N	108.7392	108.803
4	NHE	H1	7.2013	7.195
4	NHE	H2	7.5906	7.5903

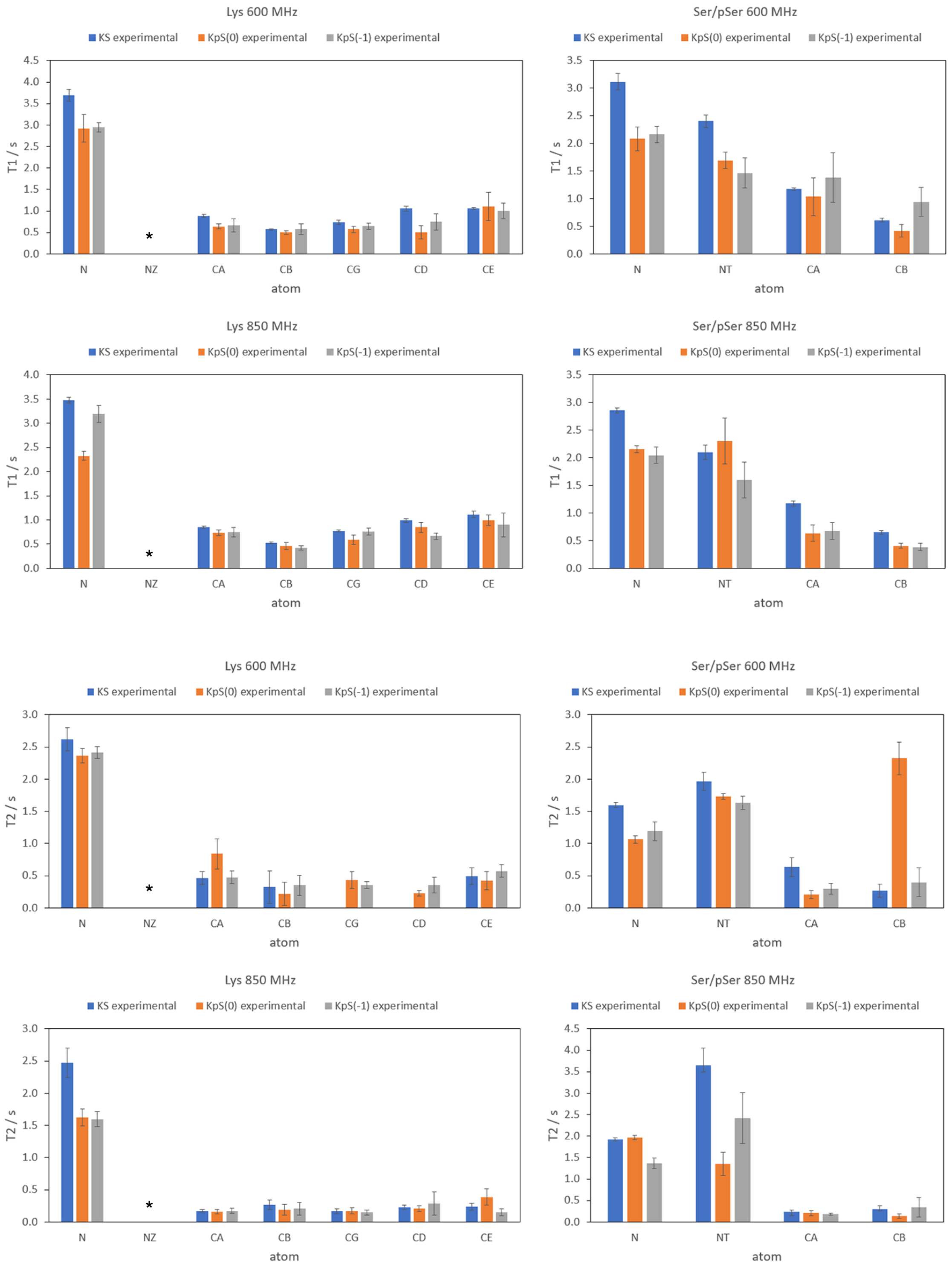

Figure S9: Comparison of experimental NMR relaxation times T_1 and T_2 between the unphosphorylated KS as Ac-K$^+$-S-NH$_2$ and the phosphorylated KpS as Ac-K$^+$pS^0-NH$_2$ and Ac-K$^+$pS$^-$-NH$_2$. For atom name mapping consult Figure 4 in the main text. The * in the plot marks atoms which cannot be measured experimentally. The error bars denote standard errors from the peak fit of the experimental resonances according to Equation 7 in the main text. The experiment was conducted at 298 K.

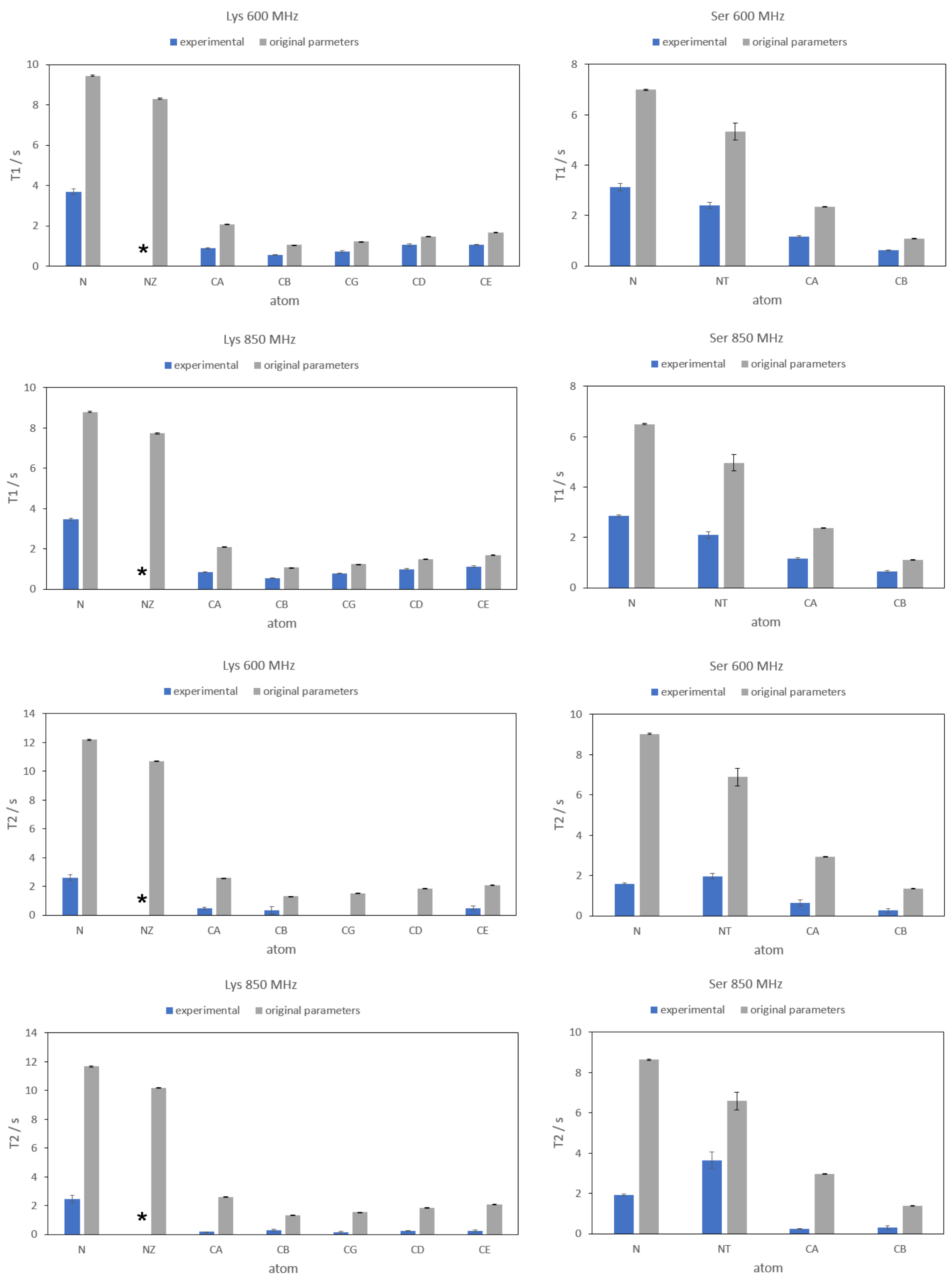

Figure S10: Comparison of NMR relaxation times T_1 and T_2 between experiment and simulation (original CHARMM36m parameters, CHARMM TIP3P water model) for Ac-K$^+$-S-NH_2. For mapping of the atom names, consult Figure 4 in the main text. The * in the plot marks atoms which cannot be measured experimentally. The error bars denote standard error of the mean estimated over 3 independent dipeptides in MD simulations and standard errors from the peak fit of the experimental resonances according to Equation 7 in the main text. The experiment and simulation were conducted at 298 K.

Additional Molecular Dynamics Data for Protein Simulations

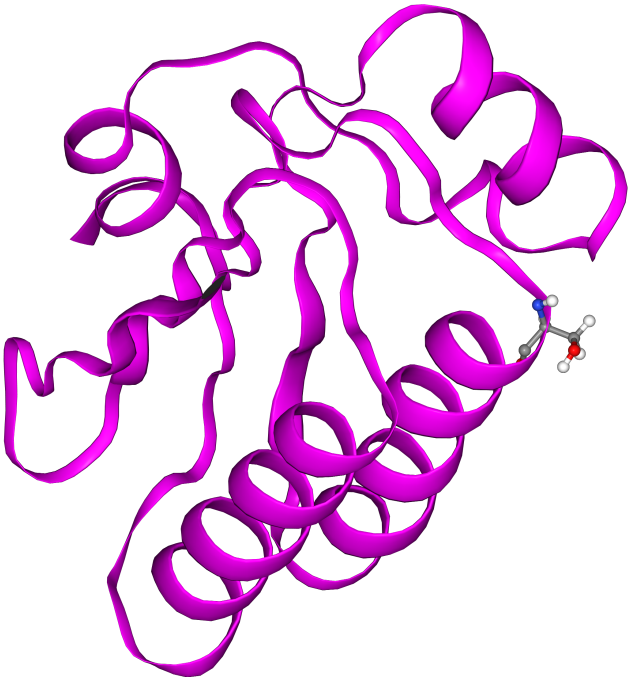

Figure S11: Cartoon depiction of the anti-sigma factor antagonist SpoIIAA wild type (1H4Y) with residue S57 shown as ball+stick.

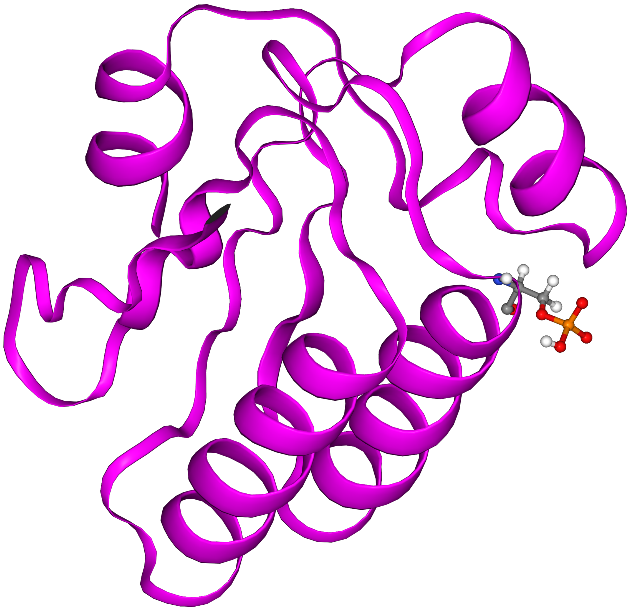

Figure S12: Cartoon depiction of the phosphorylated anti-sigma factor antagonist SpoIIAA (1H4X) with resdiue pS57 shown as ball+stick.

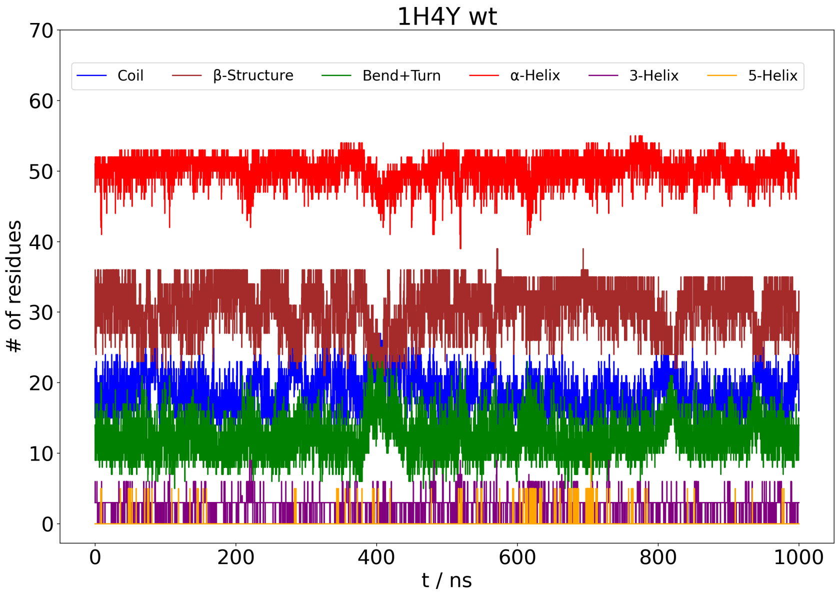

Figure S13: DSSP secondary structure analysis of the anti-sigma factor antagonist SpoIIAA in its unphosphorylated form (1H4Y wt).

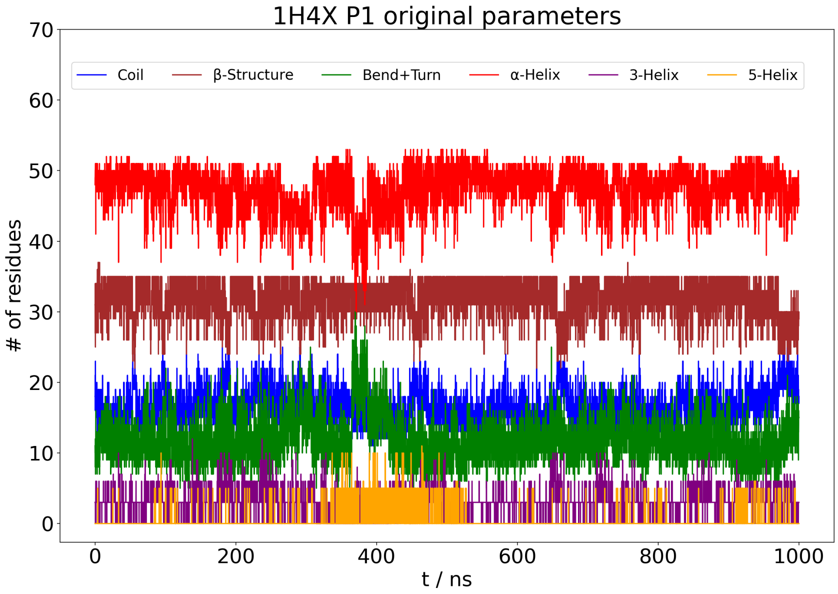

Figure S14: DSSP secondary structure analysis of the anti-sigma factor antagonist SpoIIAA in its phosphorylated form (1H4X) with a singly charged pS57 (P1) simulated with the original CHARMM36m parameters.

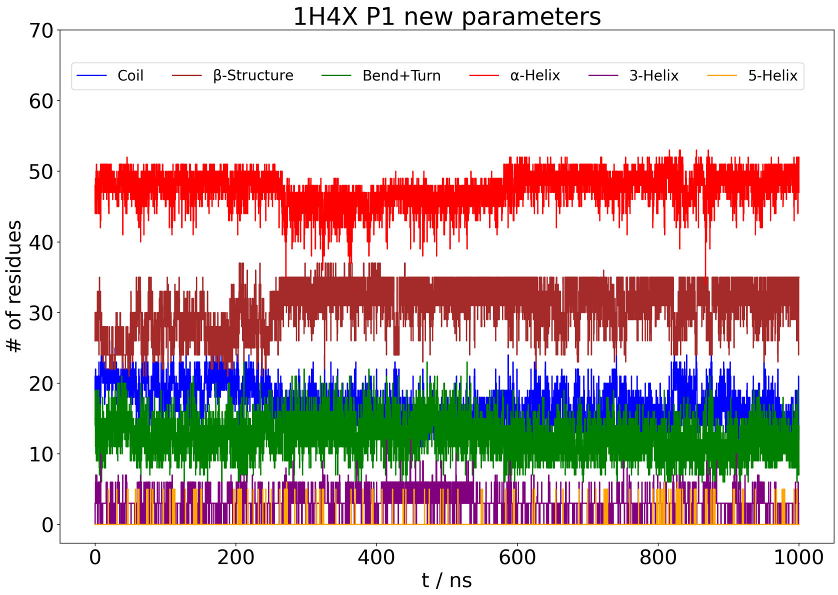

Figure S15: DSSP secondary structure analysis of the anti-sigma factor antagonist SpoIIAA in its phosphorylated form (1H4X) with a singly charged pS57 (P1) simulated with our final MP1 parameters from Table 2 in the main text.

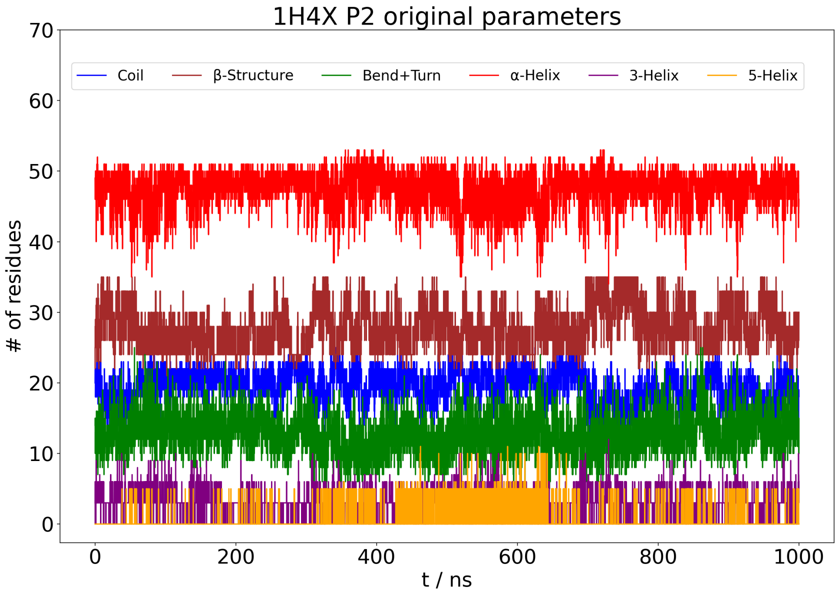

Figure S16: DSSP secondary structure analysis of the anti-sigma factor antagonist SpoIIAA in its phosphorylated form (1H4X) with a doubly charged pS57 (P2) simulated with the original CHARMM36m parameters.

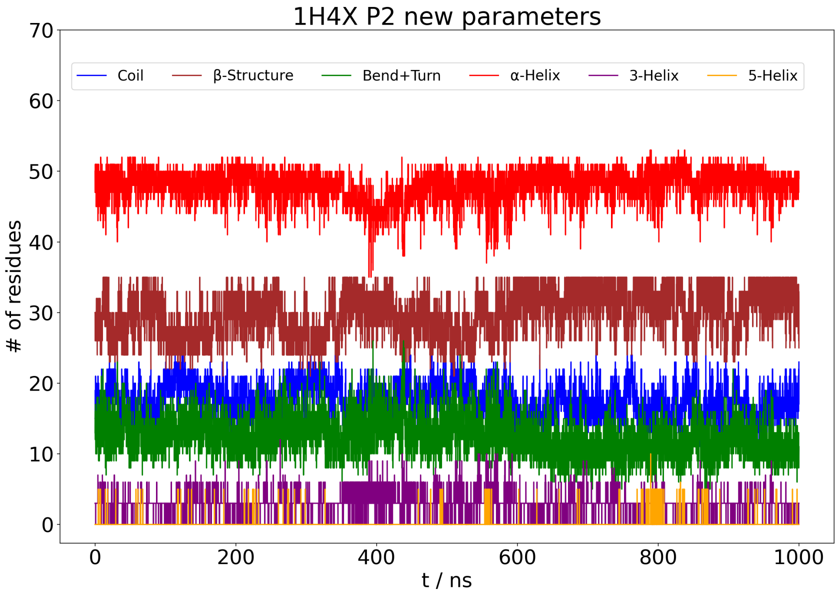

Figure S17: DSSP secondary structure analysis of the anti-sigma factor antagonist SpoIIAA in its phosphorylated form (1H4X) with a doubly charged pS57 (P2) simulated with our final parameters for MP2 from Table 2 in the main text.

Supplementary References

(1) Michaud-Agrawal, N.; Denning, E. J.; Woolf, T. B.; Beckstein, O. MDAnalysis: a toolkit for the analysis of molecular dynamics simulations. *Journal of computational chemistry* **2011**, *32*, 2319–2327.

(2) Gowers, R. J.; Linke, M.; Barnoud, J.; Reddy, T. J. E.; Melo, M. N.; Seyler, S. L.; Domanski, J.; Dotson, D. L.; Buchoux, S.; Kenney, I. M. *MDAnalysis: a Python package for the rapid analysis of molecular dynamics simulations*; 2019.

(3) Larsen, A. H.; Mortensen, J. J.; Blomqvist, J.; Castelli, I. E.; Christensen, R.; Dułak, M.; Friis, J.; Groves, M. N.; Hammer, B.; Hargus, C.; Hermes, E. D.; Jennings, P. C.; Bjerre Jensen, P.; Kermode, J.; Kitchin, J. R.; Kolsbjerg, E. L.; Kubal, J.; Kaasbjerg, K.; Lysgaard, S.; Bergmann Maronsson, J.; Maxson, T.; Olsen, T.; Pastewka, L.; Peterson, A.; Rostgaard, C.; Schiøtz, J.; Schütt, O.; Strange, M.; Thygesen, K. S.; Vegge, T.; Vilhelmsen, L.; Walter, M.; Zeng, Z.; Jacobsen, K. W. The atomic simulation environment—a Python library for working with atoms. *Journal of Physics: Condensed Matter* **2017**, *29*, 273002.

(4) Eastman, P.; Galvelis, R.; Peláez, R. P.; Abreu, C. R.; Farr, S. E.; Gallicchio, E.; Gorenko, A.; Henry, M. M.; Hu, F.; Huang, J.; Krämer, A.; Michel, J.; Mitchell, J. A.; PAnde, V. S.; Rodriguez, J. a. P.; Rodriguez-Guerra, J.; Simmonett, A. C.; Singh, S.; Swails, J.; Turner, P.; Wang, Y.; Zhang, I.; Chodera, J. D.; De Fabritiis, G.; Markland, T. E. OpenMM 8: Molecular Dynamics Simulation with Machine Learning Potentials. *The Journal of Physical Chemistry B* **2023**, *128*, 109–116.

(5) Optimization (scipy.optimize) `https://docs.scipy.org/doc/scipy/tutorial/optimize.html` (accessed 2025-11-11).

(6) Hansen, N.; Akimoto, Y.; Baudis, P. CMA-ES/pycma on Github. Zenodo, DOI:10.5281/zenodo.2559634, 2019; `https://doi.org/10.5281/zenodo.2559634`.

(7) Todorović, M.; Gutmann, M. U.; Corander, J.; Rinke, P. Bayesian inference of atomistic structure in functional materials. *Npj computational materials* **2019**, *5*, 35.

(8) Gao, F.; Han, L. Implementing the Nelder-Mead simplex algorithm with adaptive parameters. *Computational Optimization and Applications* **2012**, *51*, 259–277.

(9) Gaus, M.; Lu, X.; Elstner, M.; Cui, Q. Parameterization of DFTB3/3OB for sulfur and phosphorus for chemical and biological applications. *Journal of chemical theory and computation* **2014**, *10*, 1518–1537.

(10) Kundu, A.; Fingerhut, B. P.; Elsaesser, T. Hydration structure and dynamics of phosphoric acid and its anions—Ultrafast 2D-IR spectroscopy and ab initio molecular dynamics simulations. *The Journal of Chemical Physics* **2024**, *161*.

(11) Pribil, A. B.; Hofer, T. S.; Randolf, B. R.; Rode, B. M. Structure and dynamics of phosphate ion in aqueous solution: an ab initio QMCF MD study. *Journal of computational chemistry* **2008**, *29*, 2330–2334.

(12) Sharma, B.; Chandra, A. Ab initio molecular dynamics simulation of the phosphate ion in water: insights into solvation shell structure, dynamics, and kosmotropic activity. *The Journal of Physical Chemistry B* **2017**, *121*, 10519–10529.

(13) Raguette, L. E.; Cuomo, A. E.; Belfon, K. A.; Tian, C.; Hazoglou, V.; Witek, G.; Telehany, S. M.; Wu, Q.; Simmerling, C. phosaa14SB and phosaa19SB: updated Amber force field parameters for phosphorylated amino acids. *Journal of Chemical Theory and Computation* **2024**, *20*, 7199–7209.

(14) pKa of methylamine
`https://pubchem.ncbi.nlm.nih.gov/compound/Methylamine` (accessed 2026-03-23).

(15) pKa of methylphosphonic acid
`https://pubchem.ncbi.nlm.nih.gov/compound/Methylphosphonic-acid` (accessed 2026-03-18).
